# Supplementary material for: Novel Oleanolic Acid-Phtalimidines Tethered 1,2,3 Triazole Hybrids as Promising Antibacterial Agents: Design, Synthesis, In Vitro Experiments and In Silico Docking Studies
Source: Molecules. 2023 Jun 8;28(12):4655. doi: 10.3390/molecules28124655 (PMC10303287; doi:10.3390/molecules28124655)
Supplement: Supplementary file 1 [file molecules-28-04655-s001.zip › molecules-2432047-supplementary.pdf]

Supporting Information

For

Novel Oleanolic acid-Phtalimidines tethered 1,2,3-triazole hybrids as promising antibacterial agents: Design, synthesis, *in vitro* experiments and *in silico* docking studies

Ghofrane Lahmadi[1,2], Mabrouk Horchani[2], Amal Dbeibia[3], Abdelkarim Mahdhi[3], Anis Romdhane[2], Ata Martin Lawson[1], Adam Daich[1], Abdel Halim Harrath[4], Hichem Ben Jannet[2,*], Mohamed Othman [1,*]

[1]Normandie Univ., UNILEHAVRE, URCOM, 76600 Le Havre, France. UR 3221, INC3M, FR-CNRS 3038, UFR ST, BP: 1123, 25 rue Philippe Lebon, F-76063 Le Havre Cedex, France.

[2]Laboratory of Heterocyclic Chemistry, LR11ES39, Faculty of Science of Monastir, University of Monastir, Avenue of Environment, 5019 Monastir, Tunisia.

[3]Laboratory of Analysis, Treatment and Valorization of Pollutants of the environment and Products. Faculty of Pharmacy, University of Monastir, Tunisia.

[4]College of Science, Departmenet of Zoology, King Saud University, Riyad, 11451, Saudi Arabia.

*Corresponding authors:

Pr. Mohamed OTHMAN. Email: mohamed.othman@univ-lehavre.fr

Pr. Hichem BEN JANNET. Email: hichem.bjannet@gmail.com.

Table of contents

1. Spectral data of propargylated phtalimidines **6b**, **8g**, **(±)-13a**, **(±)-13c**, **(±)-14d**, **(±)-14c** and **(±)-17e**.

2. Copies of ^{1}H and ^{13}C NMR spectra of propargylated phtalimidines **6b**, **8g**, **(±)-13a**, **(±)-13c**, **(±)-14d**, **(±)-14c** and **(±)-17e**.

3. Copies of ^{1}H and ^{13}C NMR spectra of targeted triazoles **18**

1. Spectral data of propargylated phtalimidines **6b**, **8g**, **(±)-13a**, **(±)-13c**, **(±)-14d**, **(±)-14c** and **(±)-17e**.

1.1. *1,5,5-trimethyl-3-(prop-2-yn-1-yl)imidazolidine-2,4-dione* (**6b**)

Sodium hydride (1.1 equiv.) was slowly added to a solution of succinimide (1 equiv.) in dry DMF (10 mL). After 2 h at room temperature propargyl bromide (80% in toluene, 1.2 equiv.) was added and stirring was continued for a further 2 h under reflux. The resultant residue was concentrated under vacuum then extracted with AcOEt (3 × 20 mL). The combined organic layers were washed with brine, dried ($MgSO_4$), filtered, and evaporated in vacuo. The residue was purified by flash column chromatography using a mixture of cyclohexane / AcOEt (50 / 50) as eluent.

Yield : 87% ; **^{1}H NMR (300 MHz, CDCl$_3$)** δ_H 4.26 (d, *J* = 2.5 Hz, 2H), 2.89 (s, 3H), 2.20 (t, *J* = 2.5 Hz, 1H), 1.38 (s, 6H); **^{13}C NMR (75 MHz, CDCl$_3$)** δ_C 175.62 (C=O), 154.10 (C=O), 77.16 (C^q), 71.56 (CH), 61.57 (C^q), 28.04 (CH_2), 24.55 (CH_3), 22.02 (2 x CH_3).

***1.2.** 2-(but-3-yn-1-yl)-3-oxoisoindolin-1-yl acetate (**8g**)*

To a stirred solution of **6g** (1 equiv.) in MeOH (10 mL), was added $NaBH_4$ (4 equiv.) at 0 – 20 °C and the mixture was stirred for an hour. After completion of the reaction, saturated sodium bicarbonate solution (10 mL) was added and the organic layer was extracted with DCM (3 x 20 mL). The combined organic layers were washed with brine, dried ($MgSO_4$), filtered, and evaporated in vacuo. The crude product was purified by flash column chromatography using a mixture of cyclohexane/AcOEt (50 / 50) as eluent. Resulting product was then dissolved with DMAP (0.1 equiv.) in DCM, followed by the addition of Ac_2O (2 equiv.) and Et_3N (2 equiv.).

The reaction mixture was stirred at room temperature for 24 h. Solvent was removed under reduced pressure and the crude product was purified by flash column chromatography using a mixture of cyclohexane/AcOEt (70 / 30) as eluent.

Yield : 98% ; **^{1}H NMR (300 MHz, $CDCl_3$)** δ_H 7.82 (dt, *J* = 5.8, 1.7 Hz, 1H_{aro}), 7.63 – 7.44 (m, 3H_{aro}), 7.15 (s, 1H), 4.06 – 3.88 (m, 1H), 3.53 – 3.37 (m, 1H), 2.70 – 2.46 (m, 2H), 2.18 (s, 3H), 2.00 (t, *J* = 2.6 Hz, 1H) ; **^{13}C NMR (75 MHz, $CDCl_3$)** δ_C 171.24 (C=O), 167.98 (C=O), 141.19 (C^q), 132.67 (CH_{aro}), 131.79 (C^q), 130.45 (CH_{aro}), 124.16 (CH_{aro}), 123.77 (CH_{aro}), 81.74 (CH), 81.24 (C^q), 70.37 (CH), 39.24 (CH_2), 21.26 (CH_3), 18.58 (CH_2).

1.3. *ethyl 1-(2-ethoxy-2-oxoethyl)-3-oxo-2-(prop-2-yn-1-yl)isoindoline-1-carboxylate* ***(±)13a***

1.3.1. *Typical procedure of condensation*

To an ice chilled solution of dimethyl or diethyl *α*-bromophthalate in dry acetonitrile (10 mL) was added under argon 2 equiv. of the appropriate amine diluted in 10 mL of acetonitrile. The mixture was stirred at room temperature for 8 h. The salt that formed was removed by filtration and the filtrate was concentrated under reduced pressure. The residue was purified by flash column chromatography using a mixture of cyclohexane/AcOEt (70 / 30) as eluent.

1.3.2. *Typical procedure of alkylation*

To a mixture of phtalimidines **(±)-12a-f**, potassium carbonate (1.09 equiv.), and 20 mL of acetonitrile was added (1.2 equiv.) of the appropriate alkyl bromide. The reaction mixture was refluxed over-night. The cooled resulting suspension was filtered off. The filtrate was concentrated in vaccuo, diluted with water, and extracted with DCM (3 x 20 mL). The organic phase was dried over $MgSO_4$ and evaporated under reduced pressure. The residue was purified by flash column chromatography using a mixture of cyclohexane/AcOEt (75 / 25) as eluent.

Yield : 90% ; **^{1}H NMR (300 MHz, $CDCl_3$)** δ_H 7.84 (d, *J* = 7.3 Hz, 1H_{aro}), 7.61 – 7.46 (m, 3H_{aro}), 4.72 (dd, *J* = 17.9, 2.5 Hz, 1H), 4.37 (dd, *J* = 18.0, 2.5 Hz, 1H), 4.27 – 4.03 (m, 4H),

3.60 (d, J = 17.3 Hz, 1H), 3.11 (d, J = 17.3 Hz, 1H), 2.14 (t, J = 2.5 Hz, 1H), 1.18 (dt, J = 18.0, 7.1 Hz, 6H) ; **^{13}C NMR (75 MHz, $CDCl_3$)** δ_C 169.56 (C=O), 169.38 (C=O), 167.98 (C=O), 143.29 (C^q), 132.55 (CH_{aro}), 130.77 (C^q), 129.69 (CH_{aro}), 124.14 (CH_{aro}), 122.20 (CH_{aro}), 79.43 (C^q), 71.50 (CH), 68.59 (C^q), 62.65 (CH_2), 61.32 (CH_2), 41.10 (CH_2), 30.65 (CH_2), 14.09 (CH_3), 14.01 (CH_3).

1.4. *ethyl 1-(3-methoxybenzyl)-3-oxo-2-(prop-2-yn-1-yl)isoindoline-1-carboxylate* ***(±)-13c***

Yield : 87% ; **^{1}H NMR (300 MHz, $CDCl_3$)** δ_H 7.69 (d, J = 7.5 Hz, $1H_{aro}$), 7.56 (d, J = 4.1 Hz, $2H_{aro}$), 7.50 – 7.39 (m, $1H_{aro}$), 6.95 (t, J = 7.9 Hz, $1H_{aro}$), 6.61 (dd, J = 8.3, 2.5 Hz, $1H_{aro}$), 6.43 (d, J = 7.6 Hz, $1H_{aro}$), 6.30 (s, $1H_{aro}$), 4.53 – 4.27 (m, 2H), 4.30 – 4.06 (m, 2H), 3.75 (s, 2H), 3.55 (s, 3H), 2.34 (t, J = 2.7 Hz, 1H), 1.20 (t, J = 7.1 Hz, 3H) ; **^{13}C NMR (75 MHz, $CDCl_3$)** δ_C 170.40 (C=O), 168.55 (C=O), 159.16 (C^q), 143.87 (C^q), 135.36 (C^q), 132.00 (CH_{aro}), 131.28 (C^q), 129.26 (CH_{aro}), 129.10 (CH_{aro}), 123.94 (CH_{aro}), 122.50 (CH_{aro}), 122.36 (CH_{aro}), 115.20 (CH_{aro}), 113.05 (CH_{aro}), 78.15 (C^q), 73.34 (C^q), 72.07 (CH), 62.51 (CH_2), 55.08 (CH_3), 39.82 (CH_2), 31.44 (CH_2), 13.99 (CH_3).

1.5. *methyl 2-allyl-3-oxo-1-(prop-2-yn-1-yl)isoindoline-1-carboxylate* ***(±)-14d***

Yield : 89% ; **^{1}H NMR (300 MHz, $CDCl_3$)** δ_H 7.91 – 7.82 (m, $1H_{aro}$), 7.63 – 7.42 (m, $3H_{aro}$), 6.05 – 5.86 (m, 1H), 5.36 – 5.13 (m, 2H), 4.35 – 4.10 (m, 2H), 3.65 (s, 3H), 3.32 – 3.13 (m, 2H), 1.77 (t, J = 2.6 Hz, 1H) ; **^{13}C NMR (75 MHz, $CDCl_3$)** δ_C 170.27 (C=O), 168.83 (C=O), 143.15 (C^q), 133.42 (CH), 132.32 (CH_{aro}), 132.00 (C^q), 129.67 (CH_{aro}), 123.97 (CH_{aro}), 121.47 (CH_{aro}), 118.06 (CH_2), 77.04 (C^q), 72.28 (CH), 69.95 (C^q), 53.24 (CH), 43.98 (CH_2), 25.20 (CH_2).

1.6. *ethyl 3-oxo-2-phenethyl-1-(prop-2-yn-1-yl)isoindoline-1-carboxylate* ***(±)-14c***

Yield : 92% ; **^{1}H NMR (300 MHz, $CDCl_3$)** δ_H 7.93 – 7.81 (m, 1H_{aro}), 7.62 – 7.50 (m, 3H_{aro}), 7.33 (d, *J* = 4.4 Hz, 4H_{aro}), 7.25 – 7.18 (m, 1H_{aro}), 4.33 – 4.01 (m, 2H), 3.90 – 3.75 (m, 1H), 3.69 – 3.54 (m, 1H), 3.28 – 2.98 (m, 4H), 1.88 (t, *J* = 2.6 Hz, 1H), 1.18 (t, *J* = 7.1 Hz, 3H) ; **^{13}C NMR (75 MHz, $CDCl_3$)** δ_C 169.67 (C=O), 169.19 (C=O), 143.04 (C^q), 139.30 (2 x C^q), 132.24 (CH_{aro}), 129.69 (CH_{aro}), 129.01 (2 x CH_{aro}), 128.70 (2 x CH_{aro}), 126.58 (CH_{aro}), 123.77 (CH_{aro}), 121.72 (CH_{aro}), 72.49 (CH), 70.71 (C^q), 62.77 (CH_2), 44.12 (CH_2), 34.78 (CH_2), 25.97 (CH_2), 14.08 (CH_3).

1.7. *2-((1,5-dimethyl-1H-pyrrol-2-yl)methyl)-3-(hydroxymethyl)-3-(prop-2-yn-1-yl)isoindolin-1-one* ***(±)-17e***

To a stirring solution of **(±)-14e** (851 mg, 2.77 mmol) in DCM (10 mL) at 0°C was added lithium borohydride (1.7 equiv.) portionwise over 10 min. The mixture was warmed to room temperature and stirred overnight. The reaction mixture was cooled to 0 °C and additional lithium borohydride (0.3 equiv.) was added. The mixture was warmed to room temperature and stirred for 48 h. The resulting mixture was concentrated in vaccuo and the residue was treated with 2M HCl (3 mL). The combined organic layers were washed with brine, dried ($MgSO_4$), filtered, and evaporated in vacuo. The mixture was extracted with DCM (3 x 20 mL) and the combined organic layers were washed with brine, dried ($MgSO_4$), filtered, and evaporated in vacuo. The residue was purified by flash column chromatography using a mixture of cyclohexane/AcOEt (50 / 50) as eluent.

Yield : 80% ; **^{1}H NMR (300 MHz, $CDCl_3$)** δ_H 7.87 (d, *J* = 7.2 Hz, 1H_{aro}), 7.62 – 7.46 (m, 3H_{aro}), 6.08 (d, *J* = 3.2 Hz, 1H_{aro}), 5.83 (d, *J* = 3.5 Hz, 1H_{aro}), 5.06 (d, *J* = 16.3 Hz, 1H), 4.66 (d, *J* = 16.3 Hz, 1H), 3.75 (s, 2H)., 3.48 (s, 3H), 2.72 (t, *J* = 2.7 Hz, 1H), 2.17 (s, 3H), 1.78 (t, *J* = 2.6 Hz) .

2. Copies of ^{1}H and ^{13}C NMR spectra of propargylated phtalimidines **6b**, **8g**, **(±)-13a**, **(±)-13c**, **(±)-14d**, **(±)-14c** and **(±)-17e**.

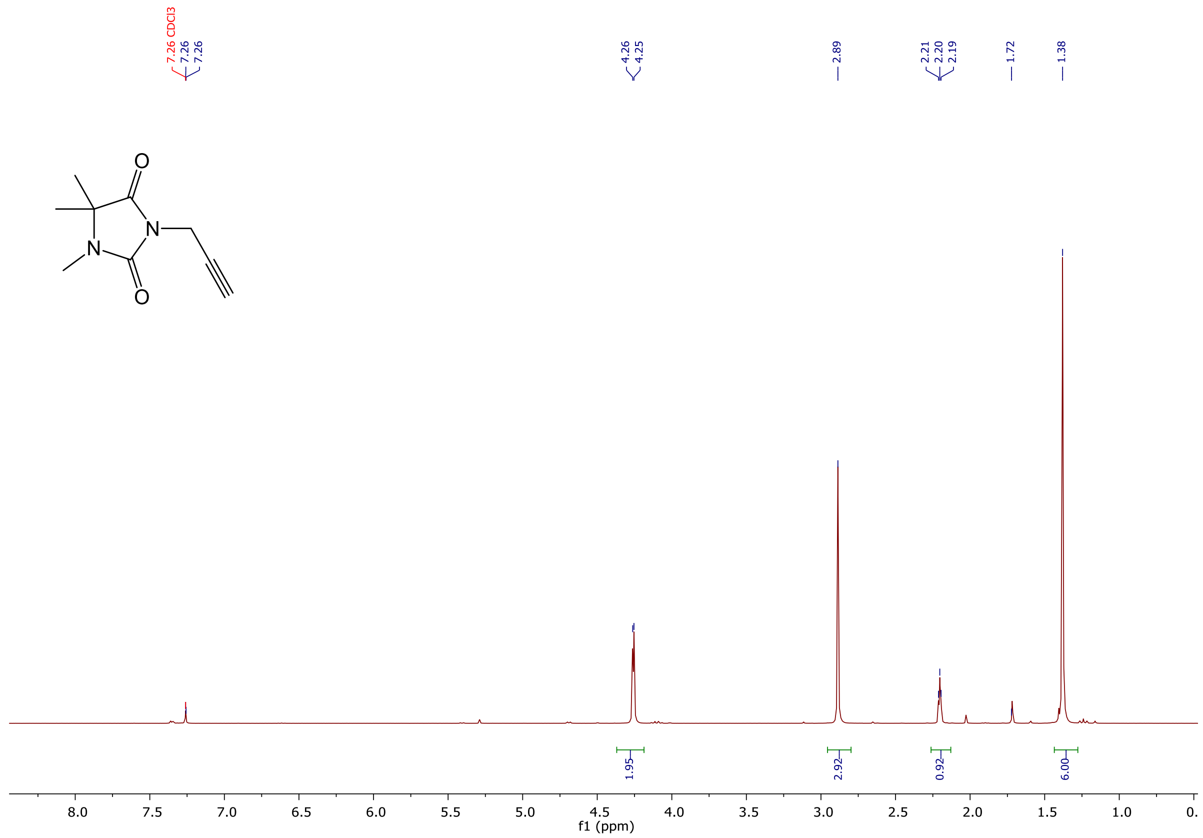

^{1}H NMR spectrum of compound **6b**

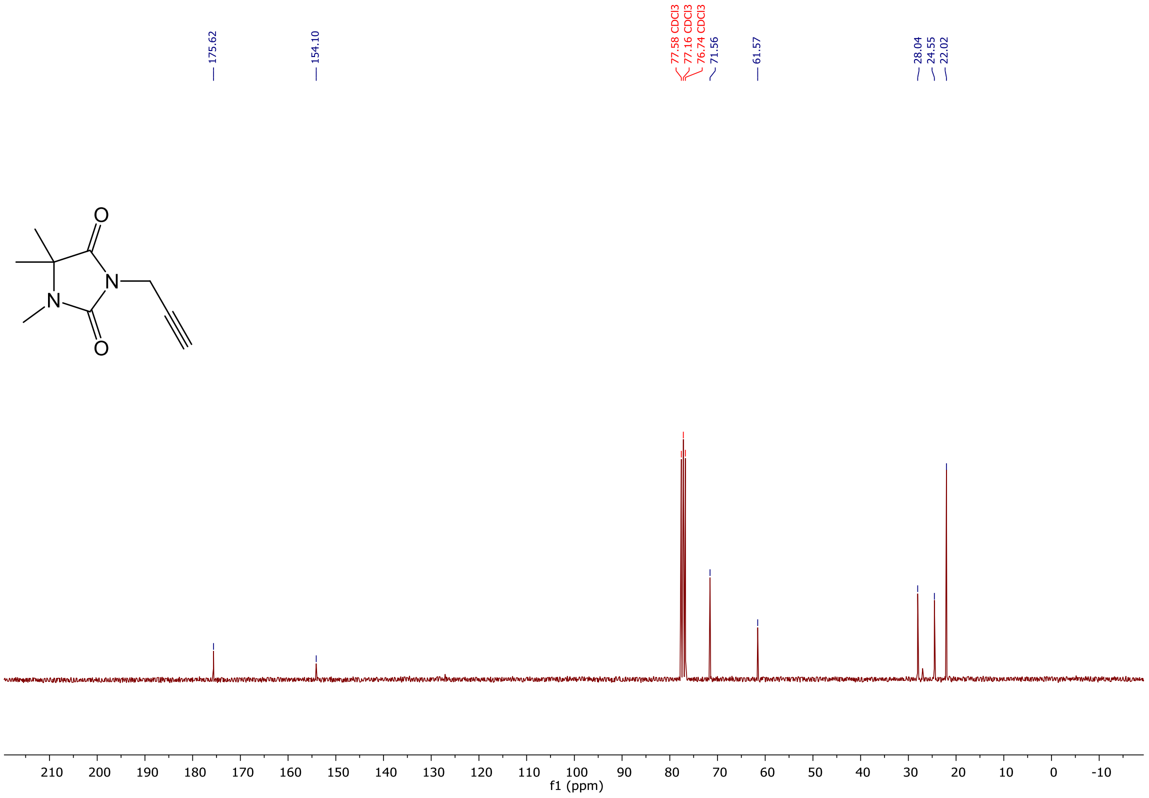

^{13}C NMR spectrum of compound **6b**

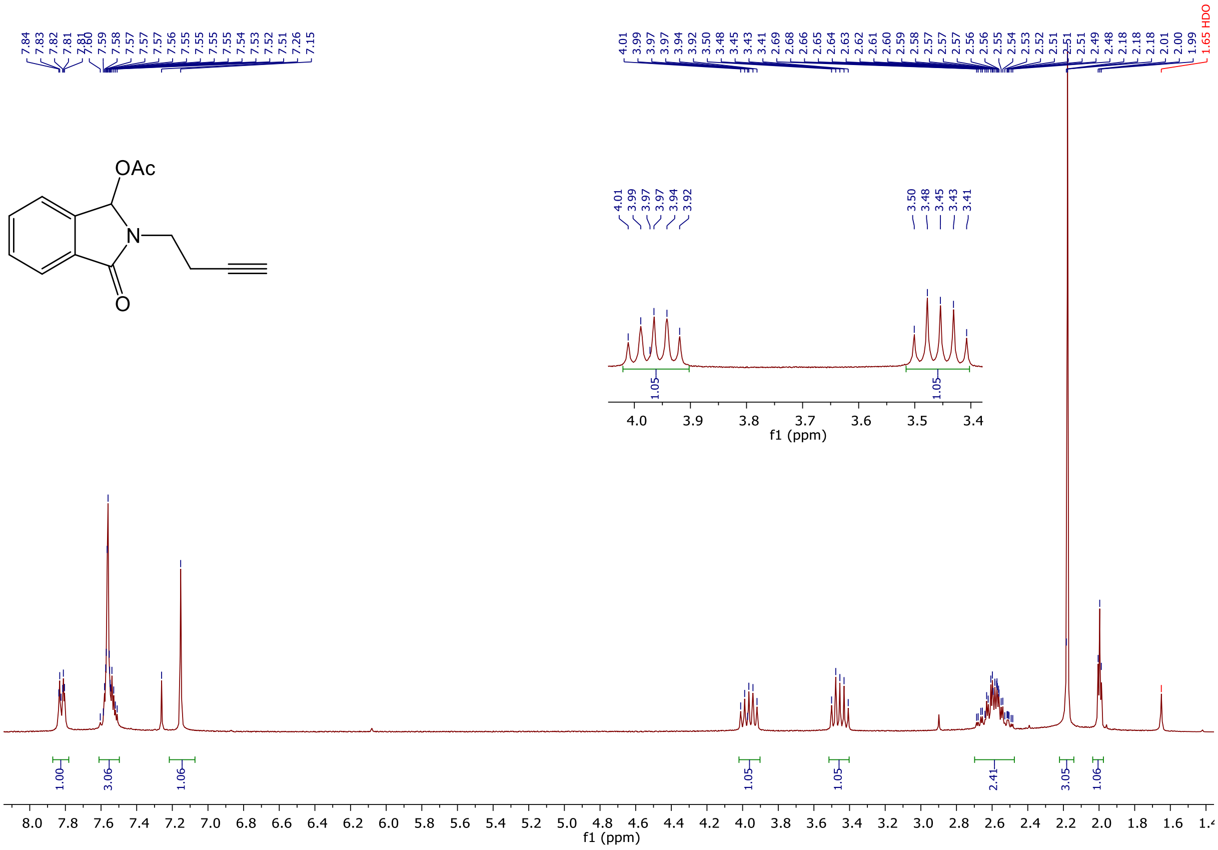

^{1}H NMR spectrum of compound **8g**

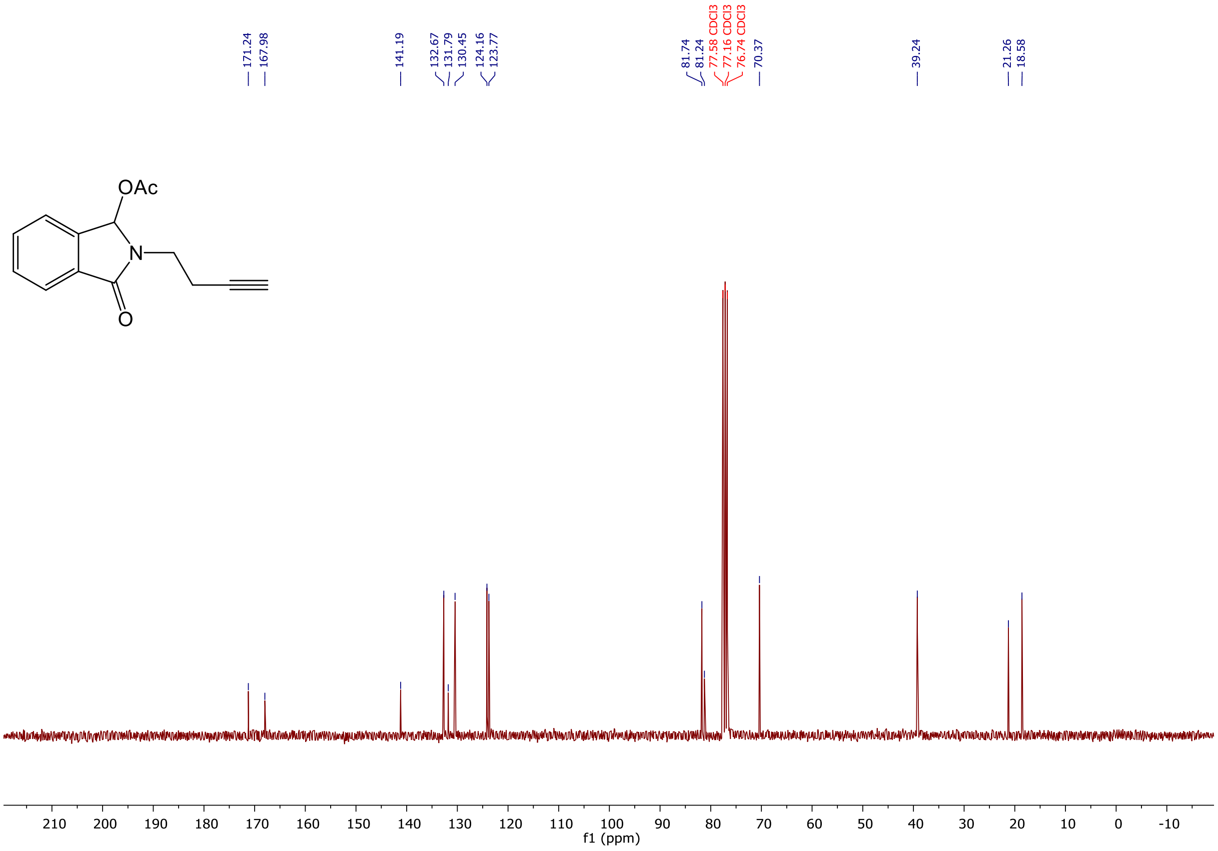

^{13}C NMR spectrum of compound **8g**

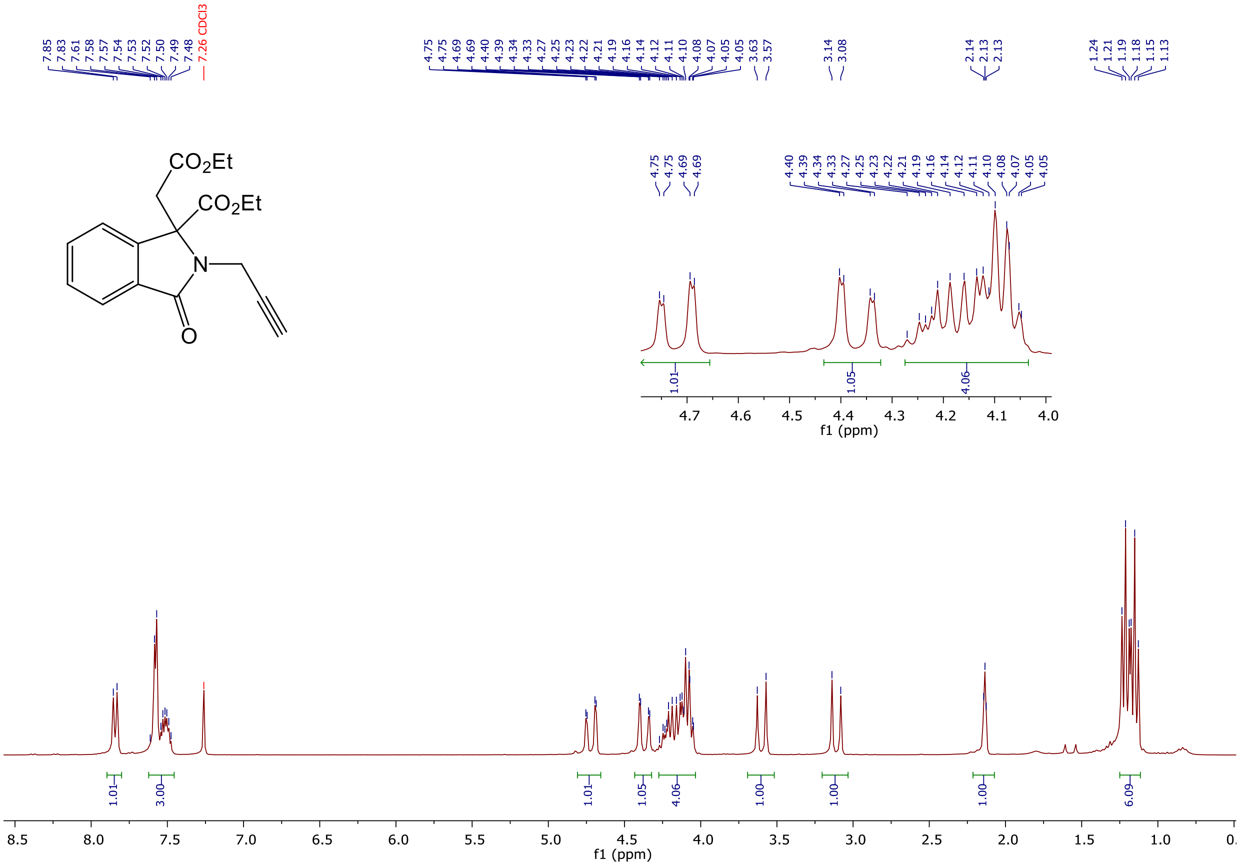

^{1}H NMR spectrum of compound **(±)-13a**

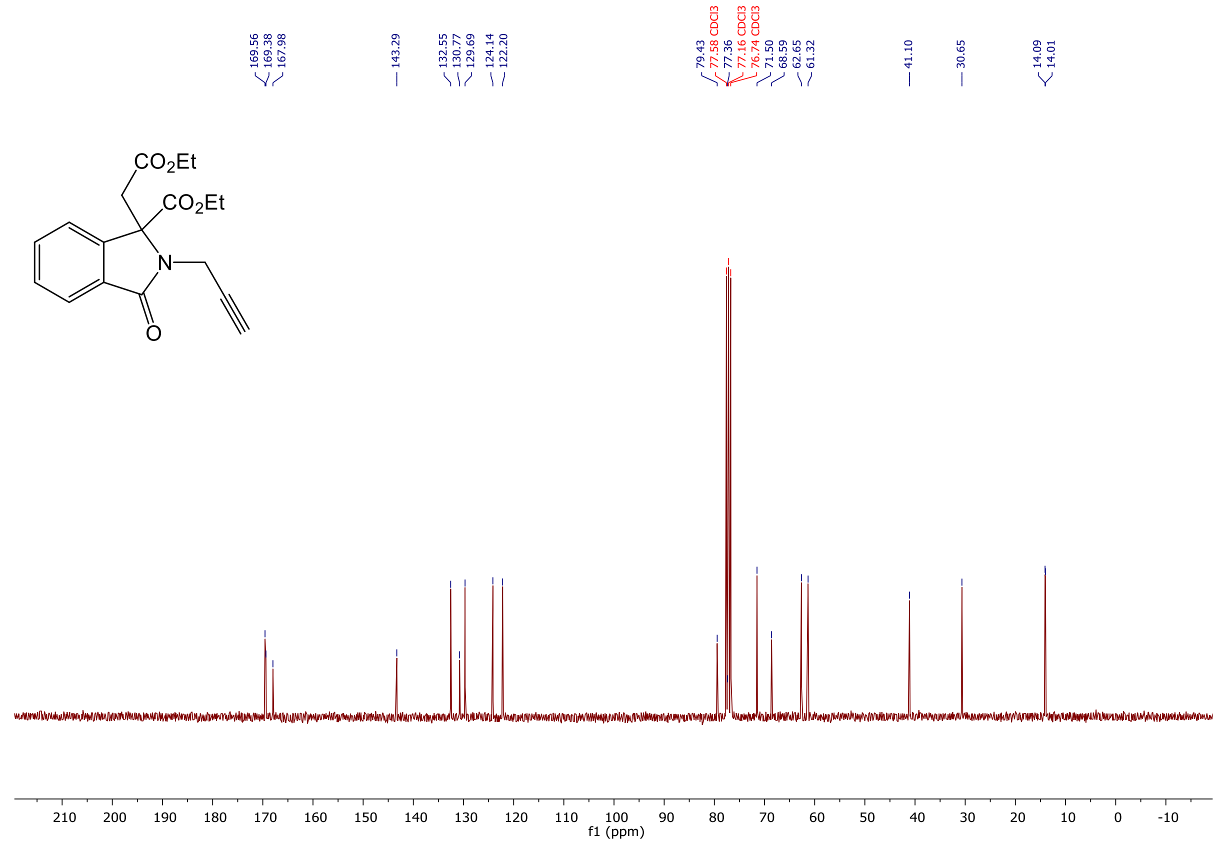

^{13}C NMR spectrum of compound **(±)-13a**

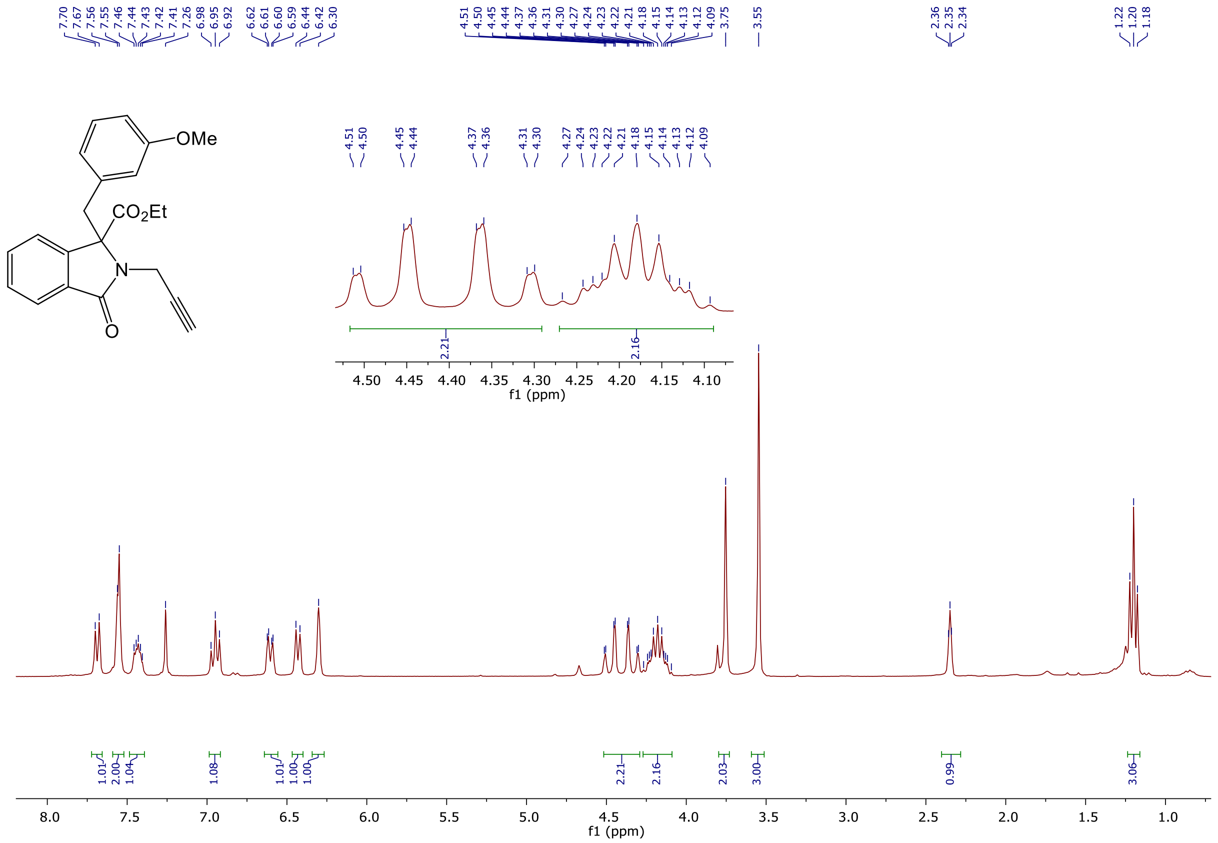

^{1}H NMR spectrum of compound **(±)-13c**

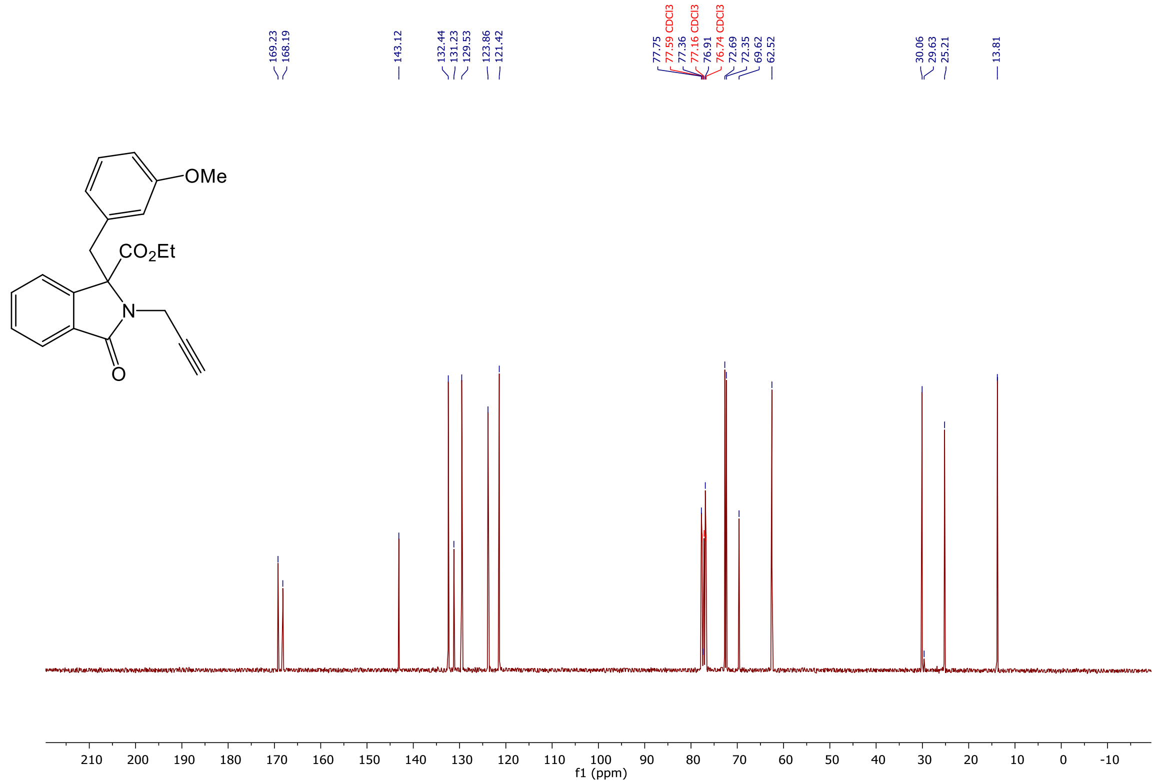

^{13}C NMR spectrum of compound **(±)-13c**

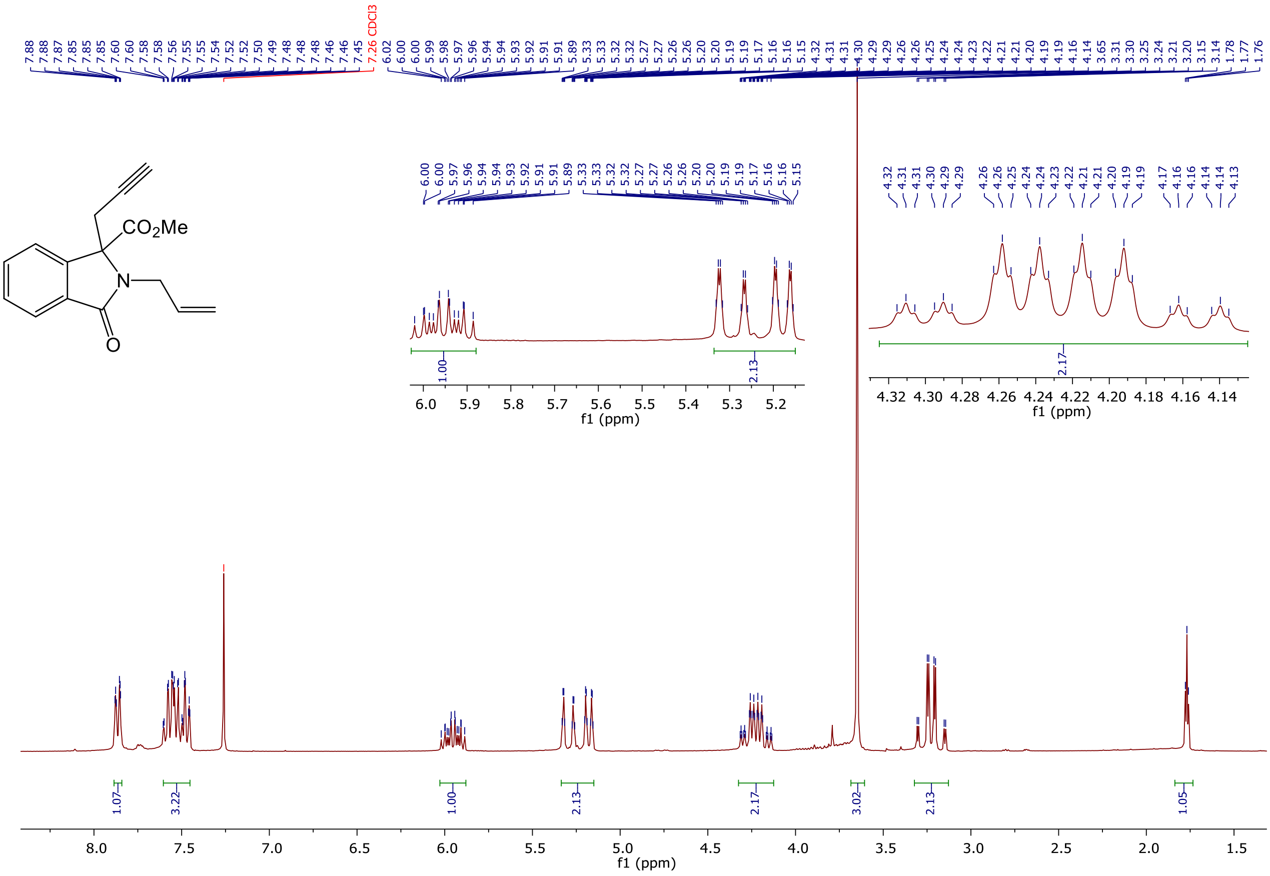

^{1}H NMR spectrum of compound **(±)-14d**

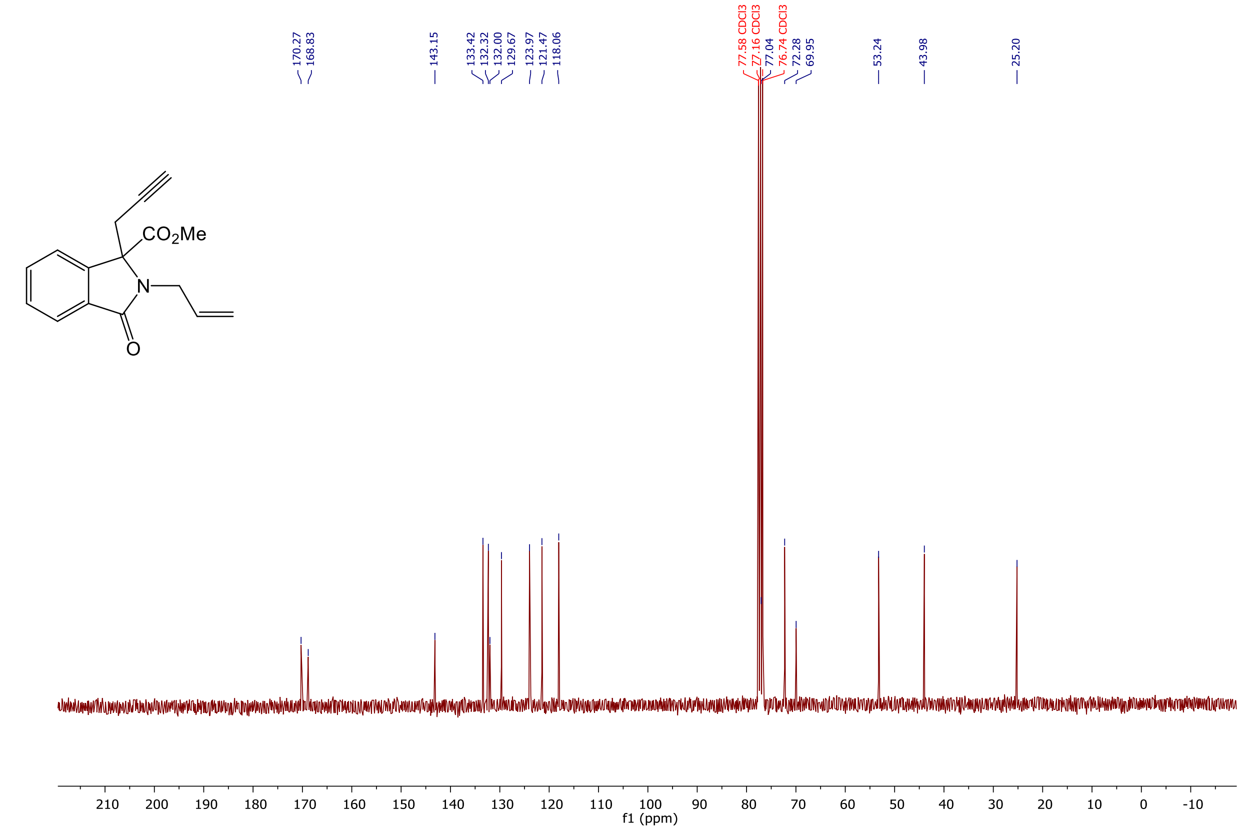

^{13}C NMR spectrum of compound **(±)-14d**

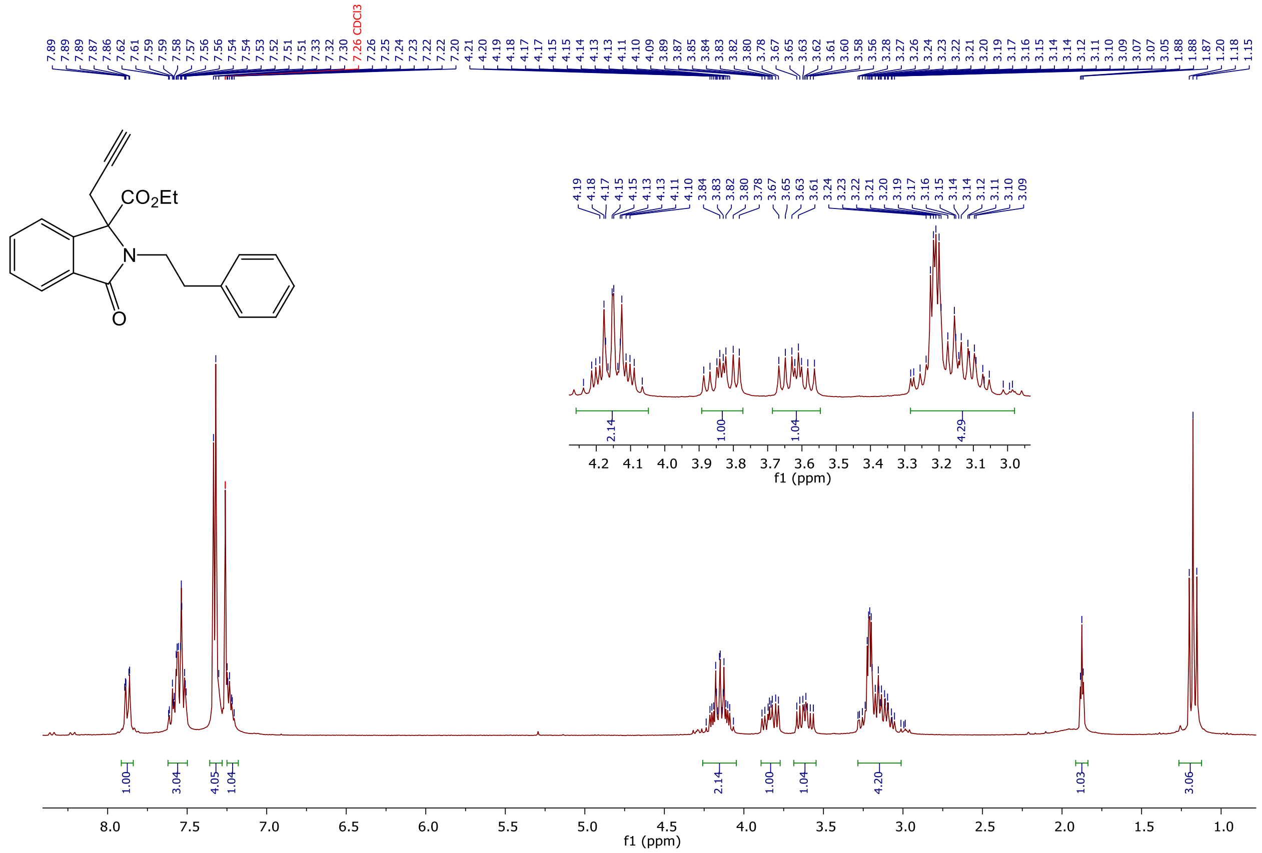

^{1}H NMR spectrum of compound **(±)-14c**

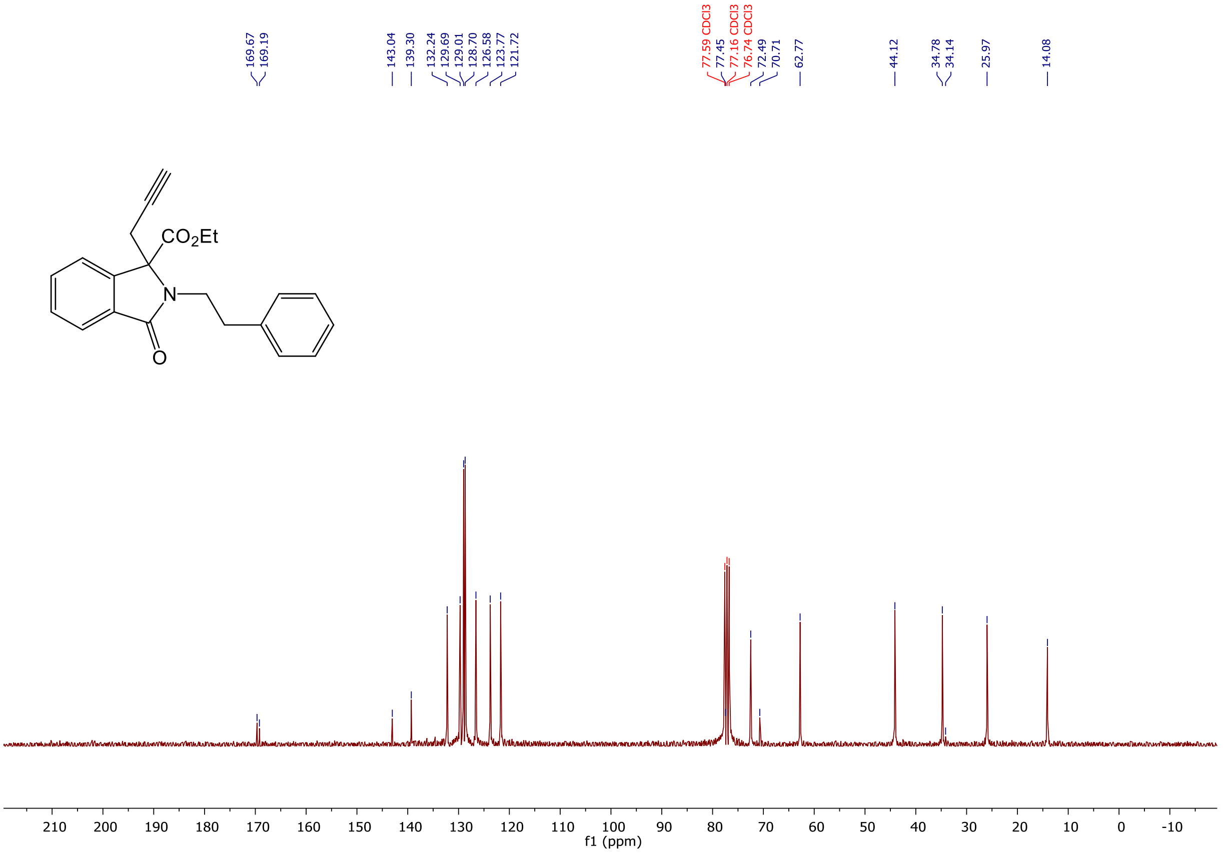

^{13}C NMR spectrum of compound **(±)-14c**

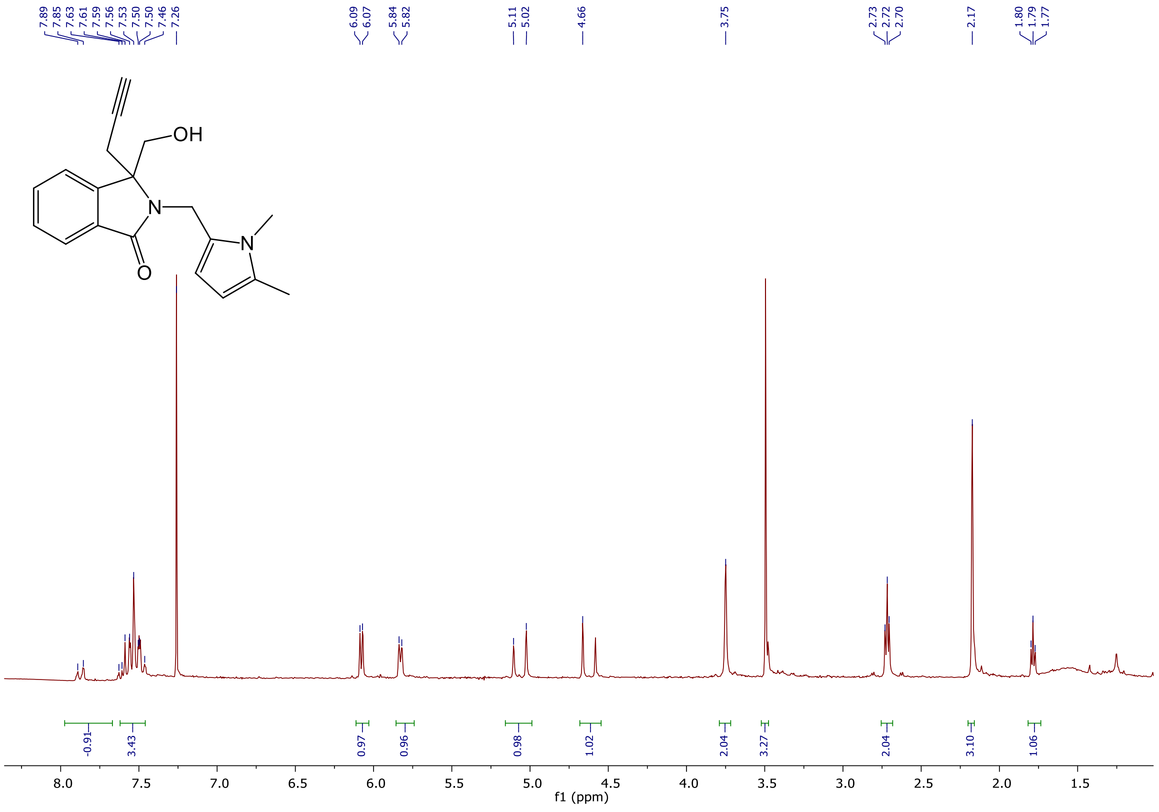

^{1}H NMR spectrum of compound **(±)-17e**

3. Copies of ^{1}H and ^{13}C NMR spectra of targeted triazoles **18**

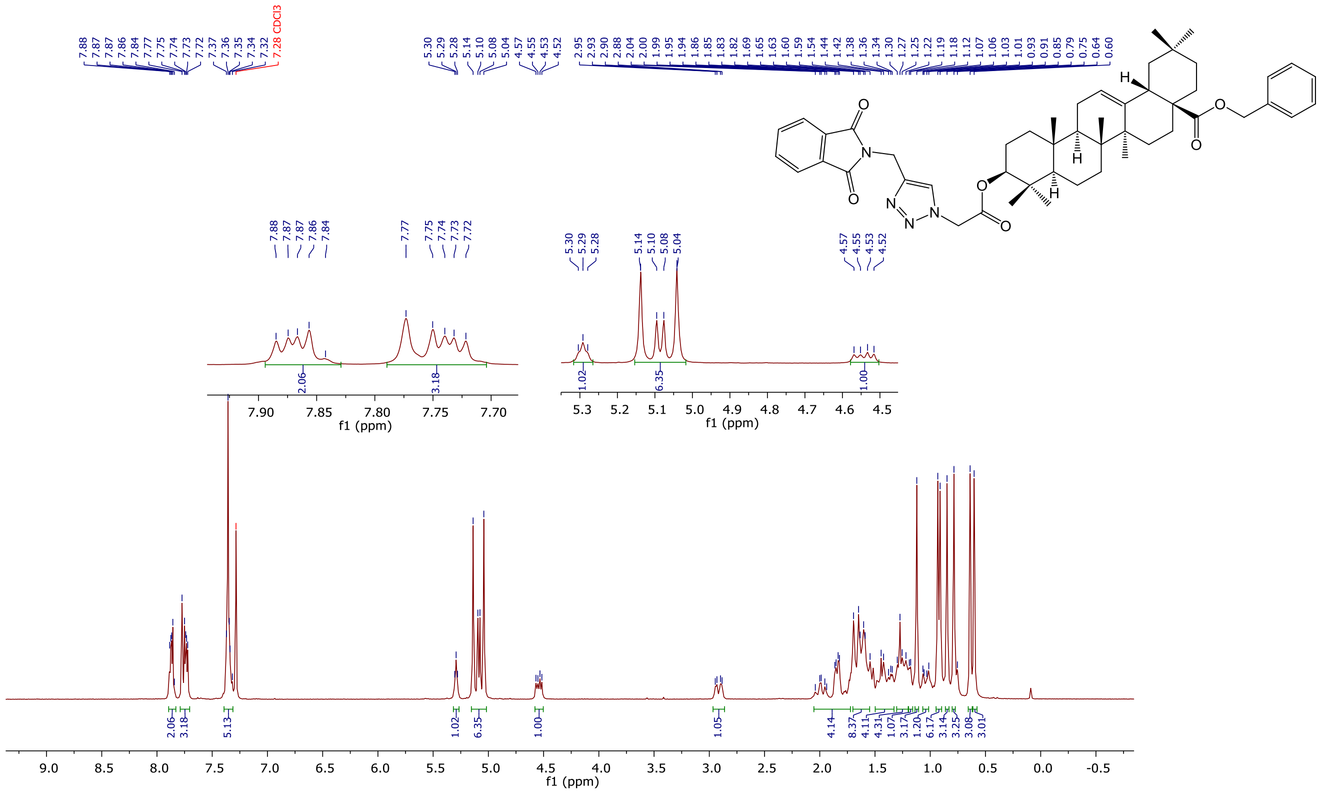

^{1}H NMR spectrum of compound **(18a)**

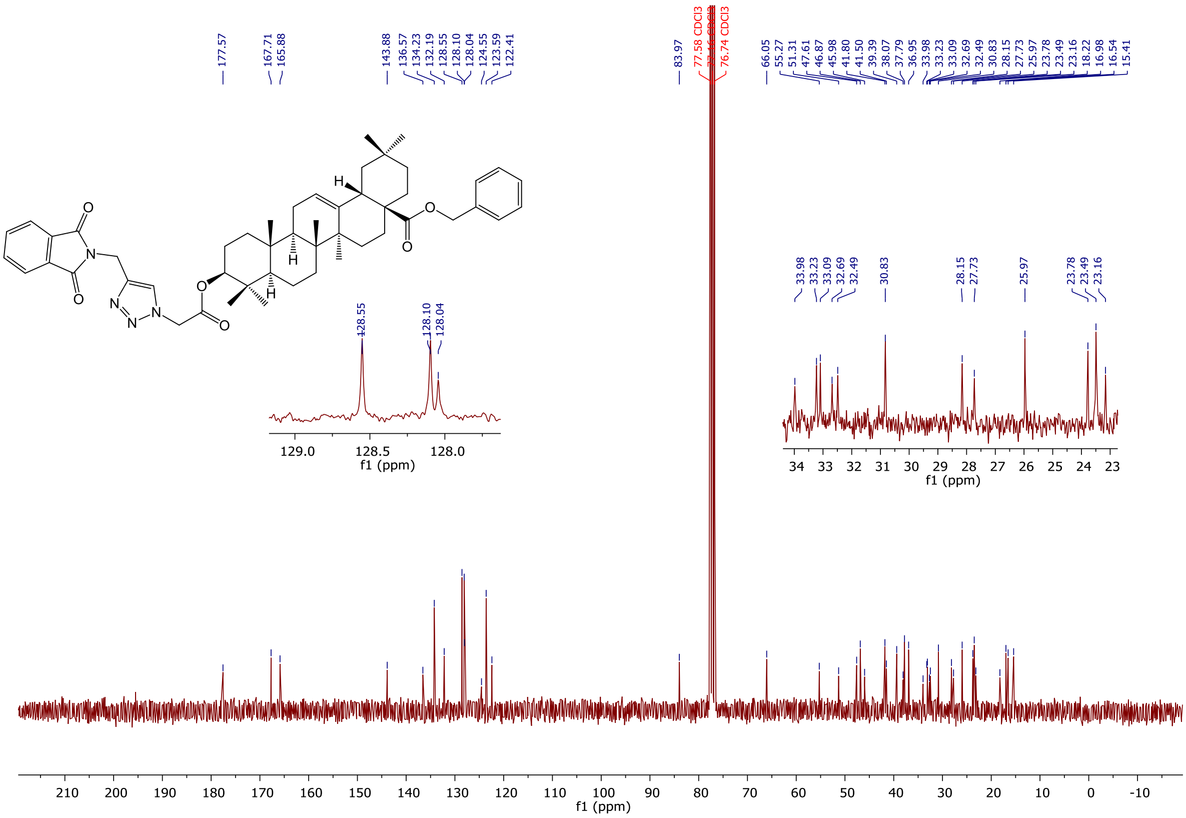

^{13}C NMR spectrum of compound **(18a)**

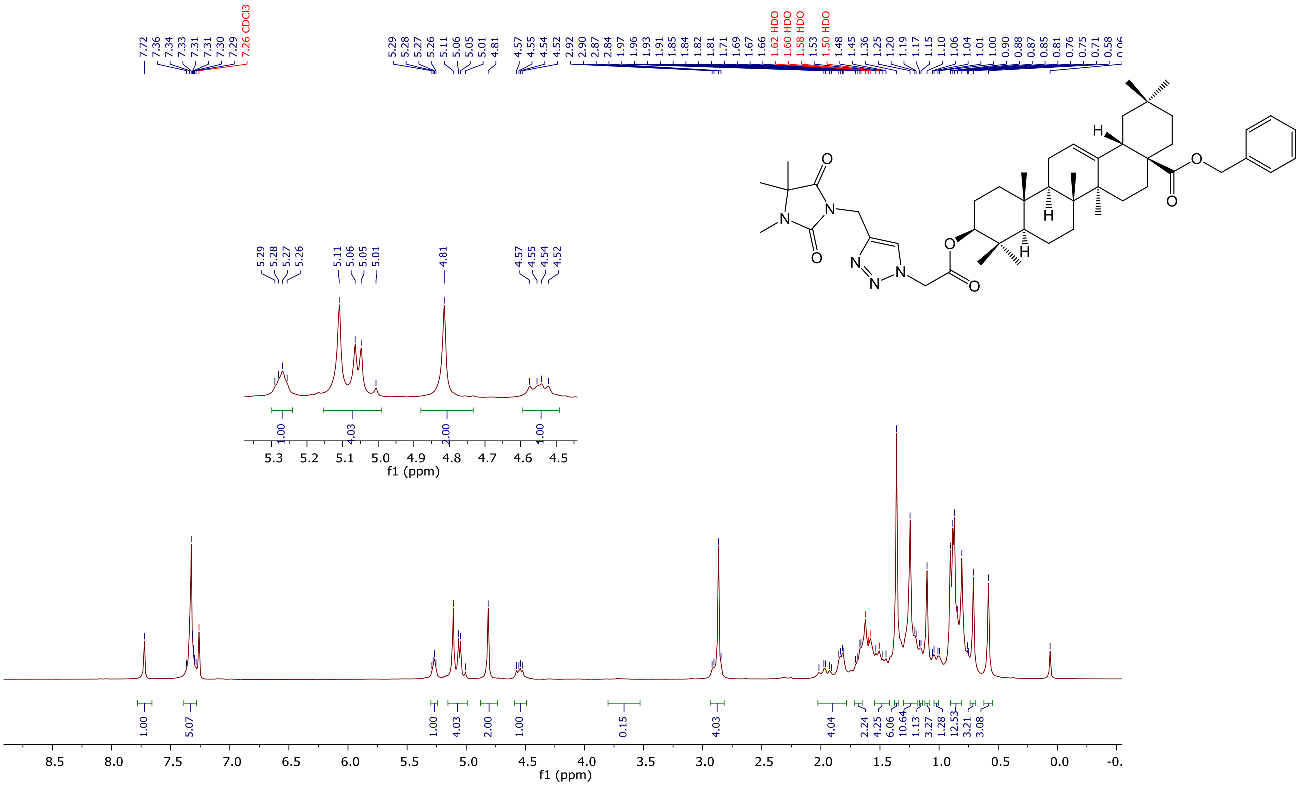

^{1}H NMR spectrum of compound **(18b)**

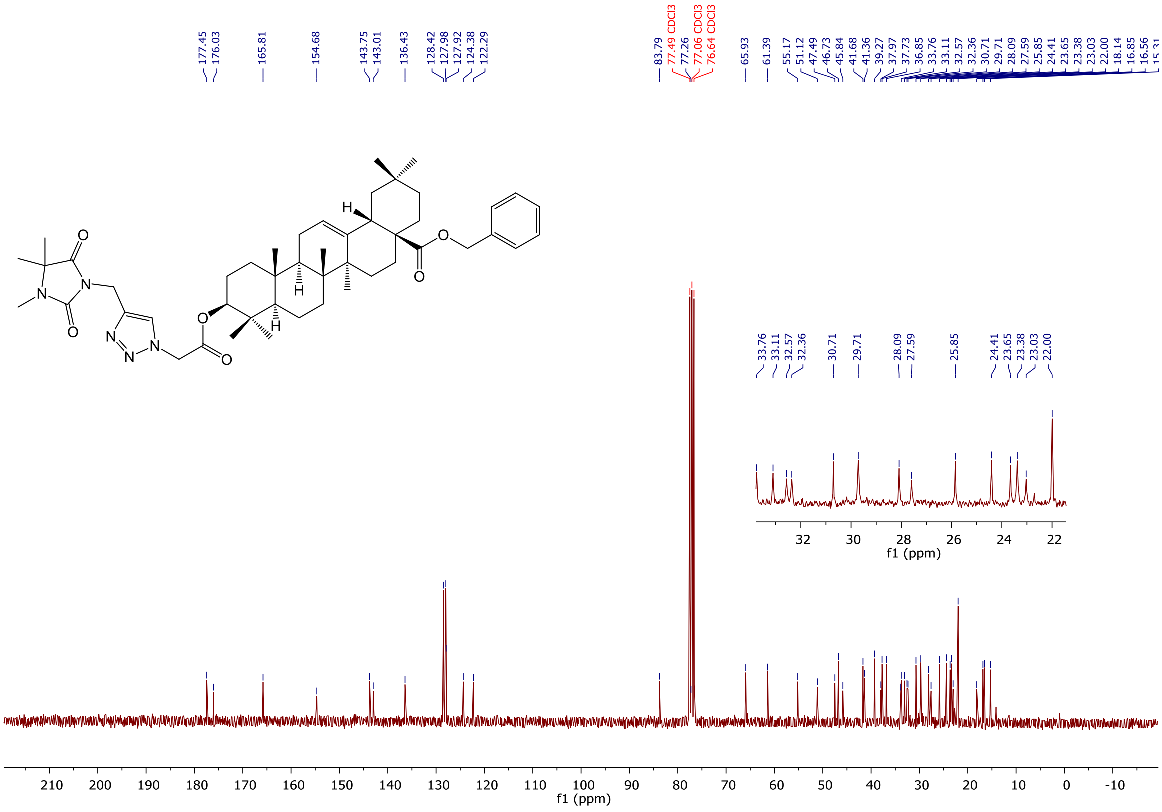

^{13}C NMR spectrum of compound **(18b)**

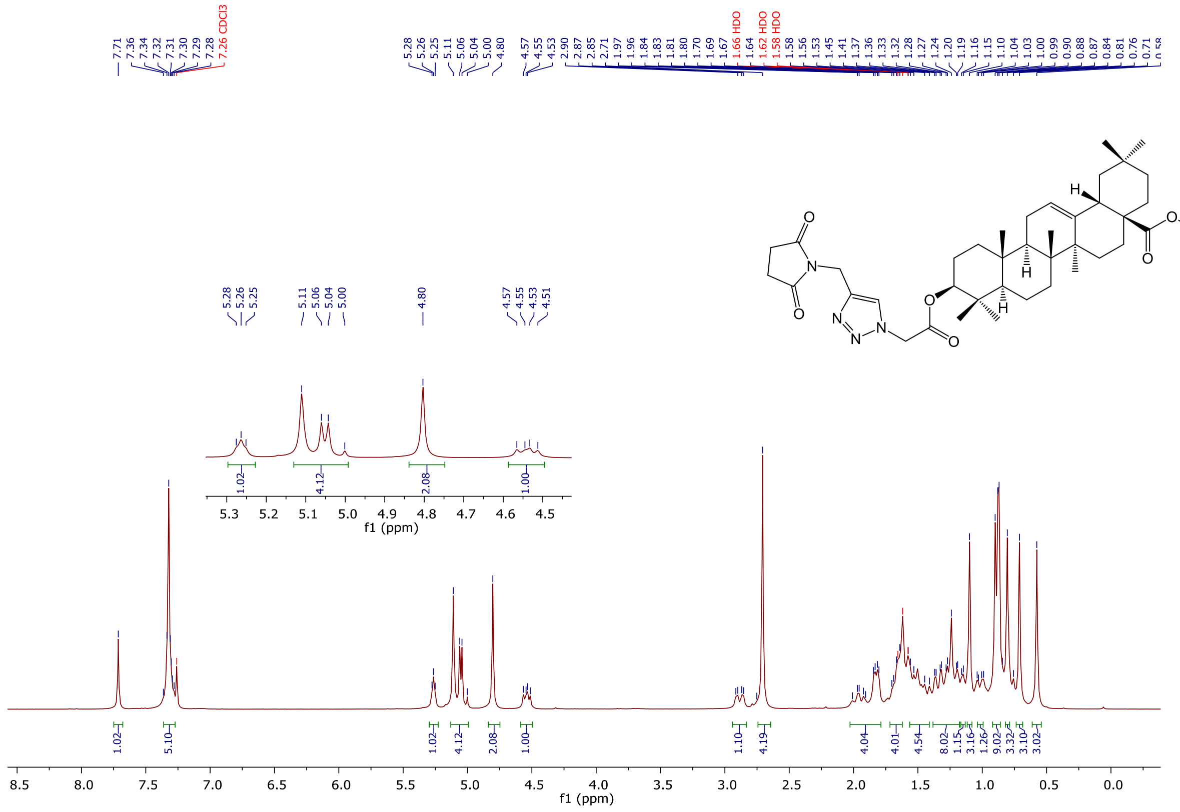

^{1}H NMR spectrum of compound **(18c)**

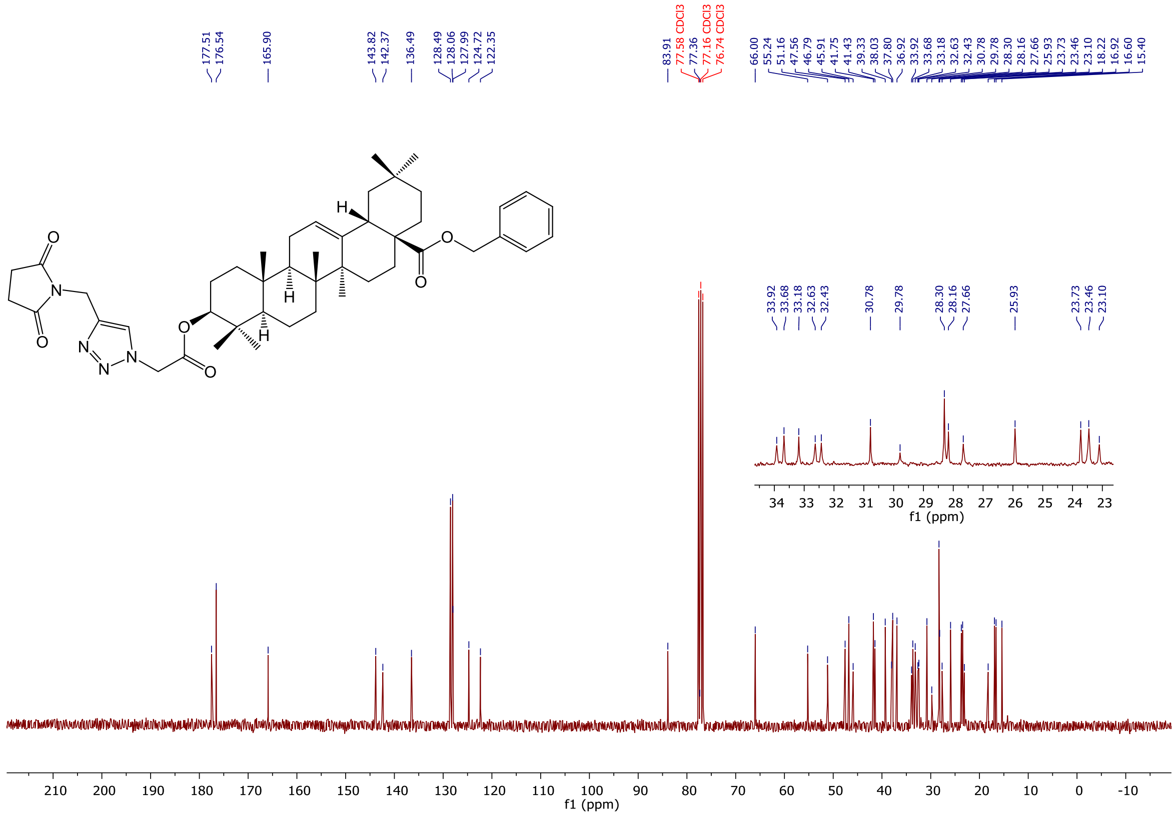

^{13}C NMR spectrum of compound **(18c)**

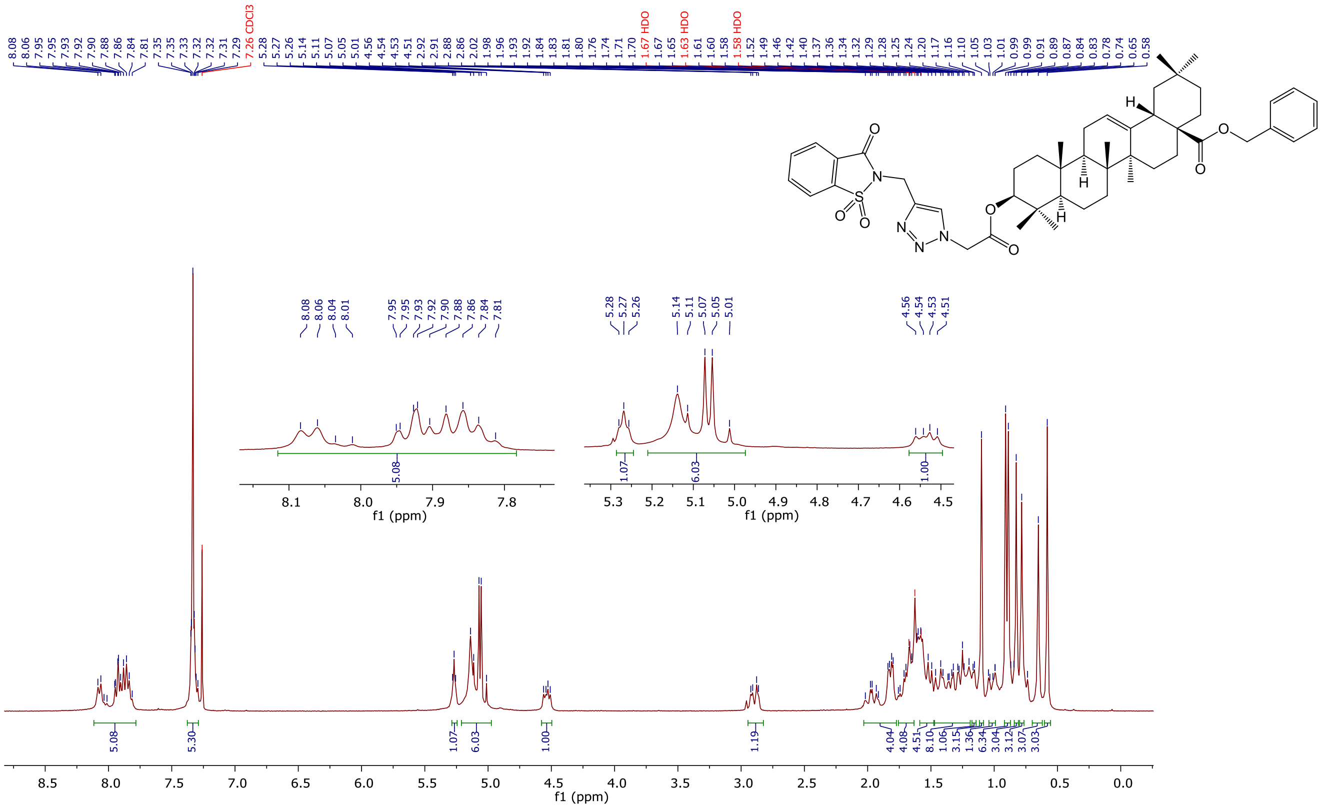

^{1}H NMR spectrum of compound **(18d)**

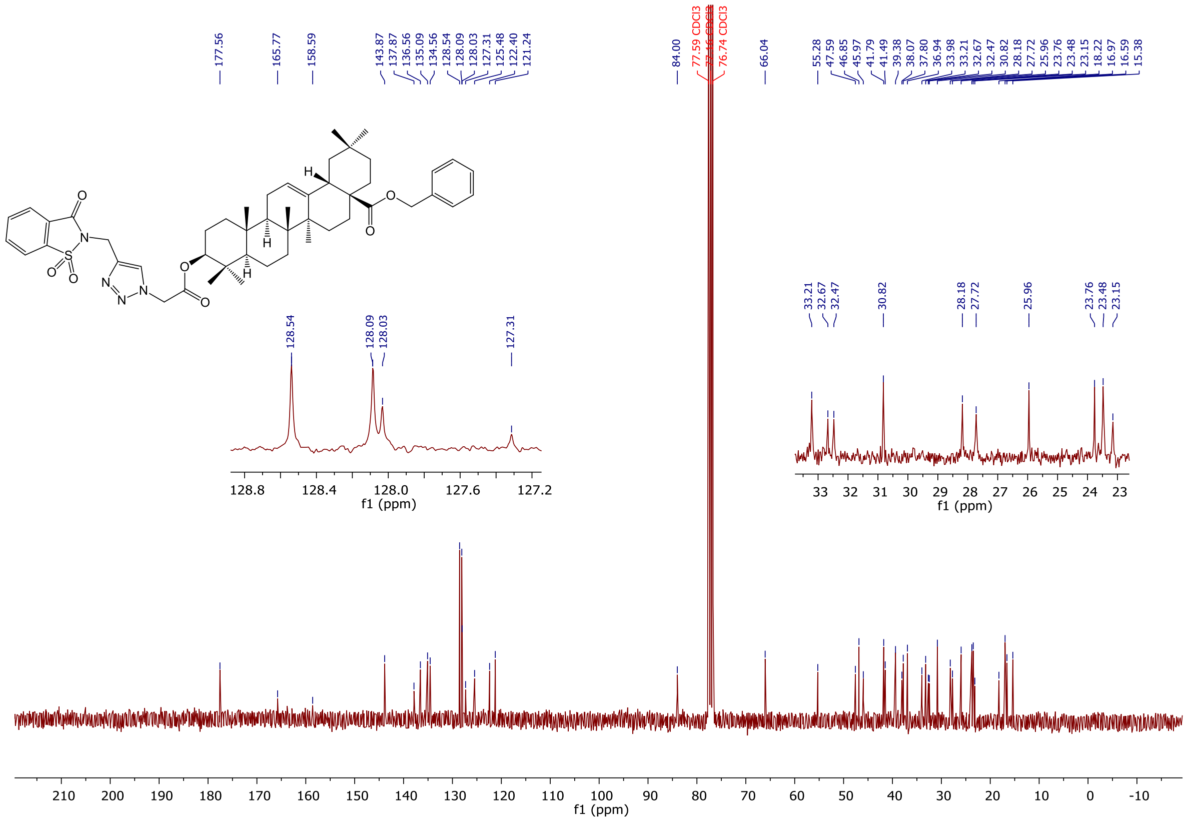

^{13}C NMR spectrum of compound **(18d)**

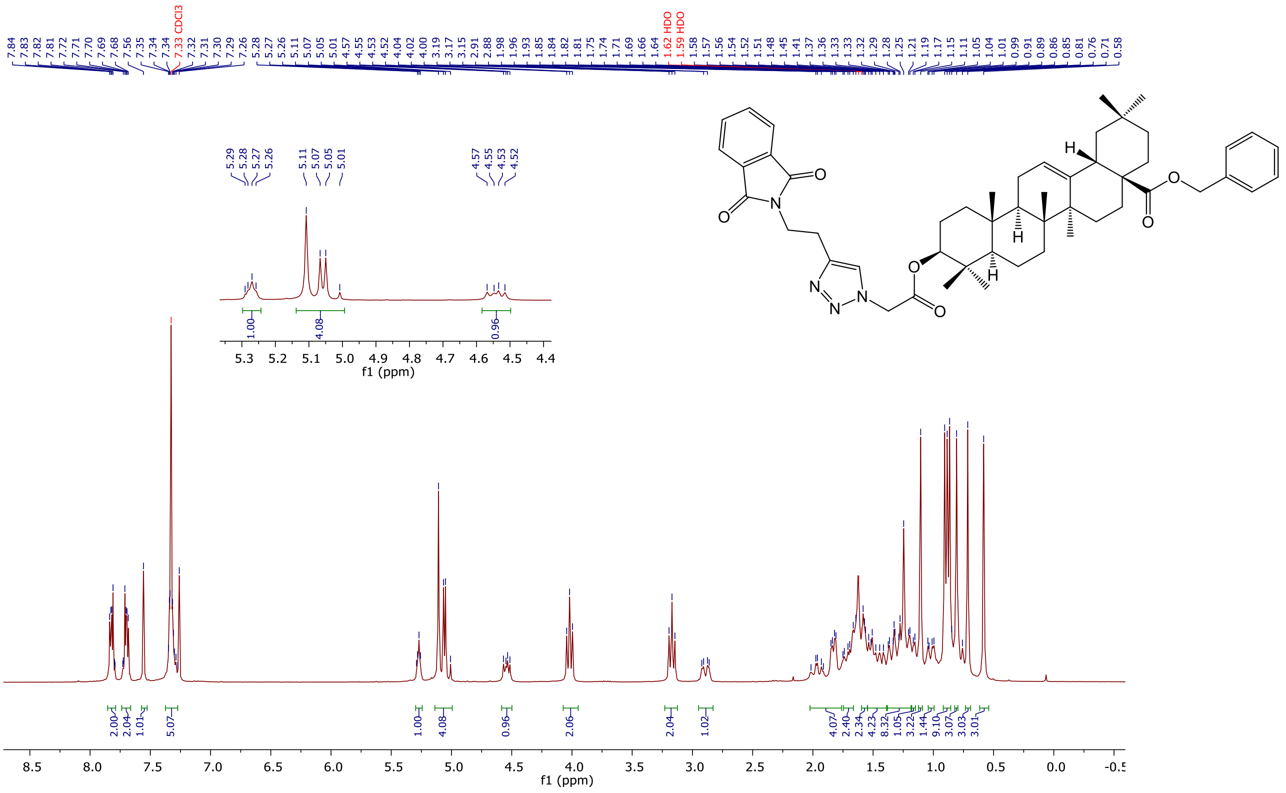

^{1}H NMR spectrum of compound **(18e)**

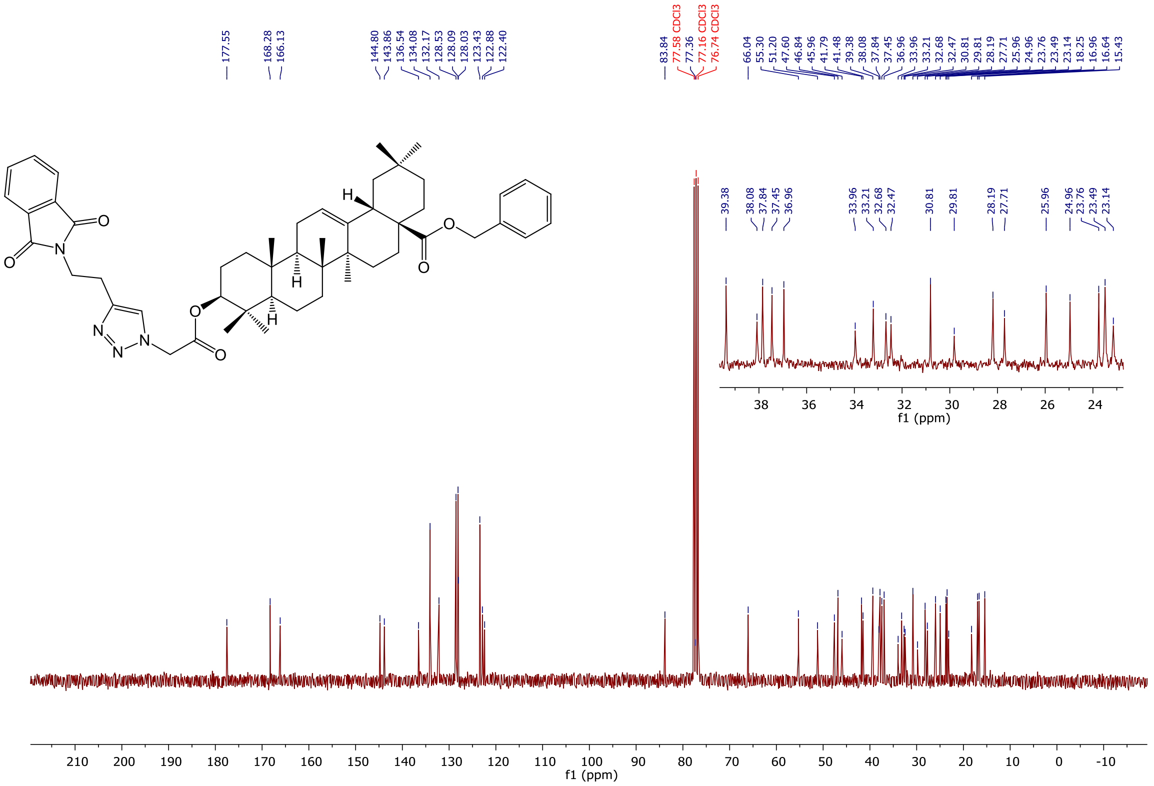

^{13}C NMR spectrum of compound **(18e)**

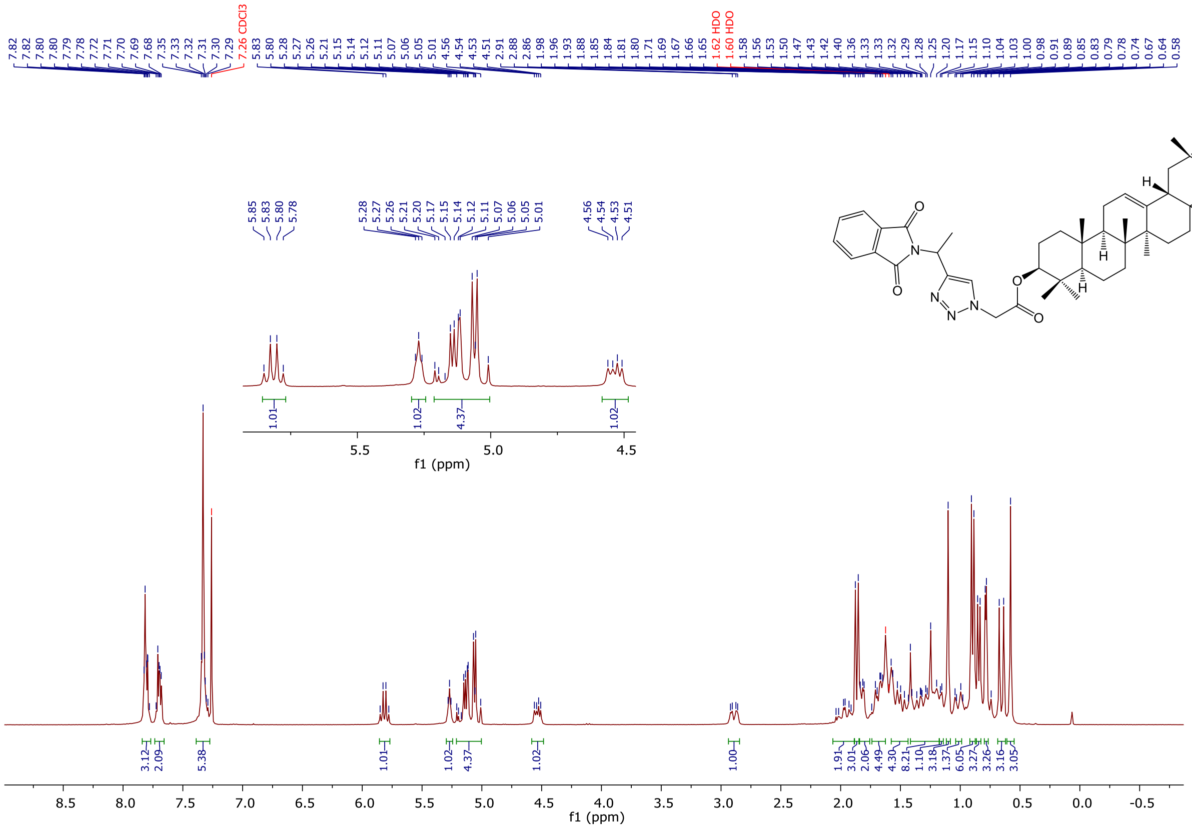

^{1}H NMR spectrum of compound **(18f)**

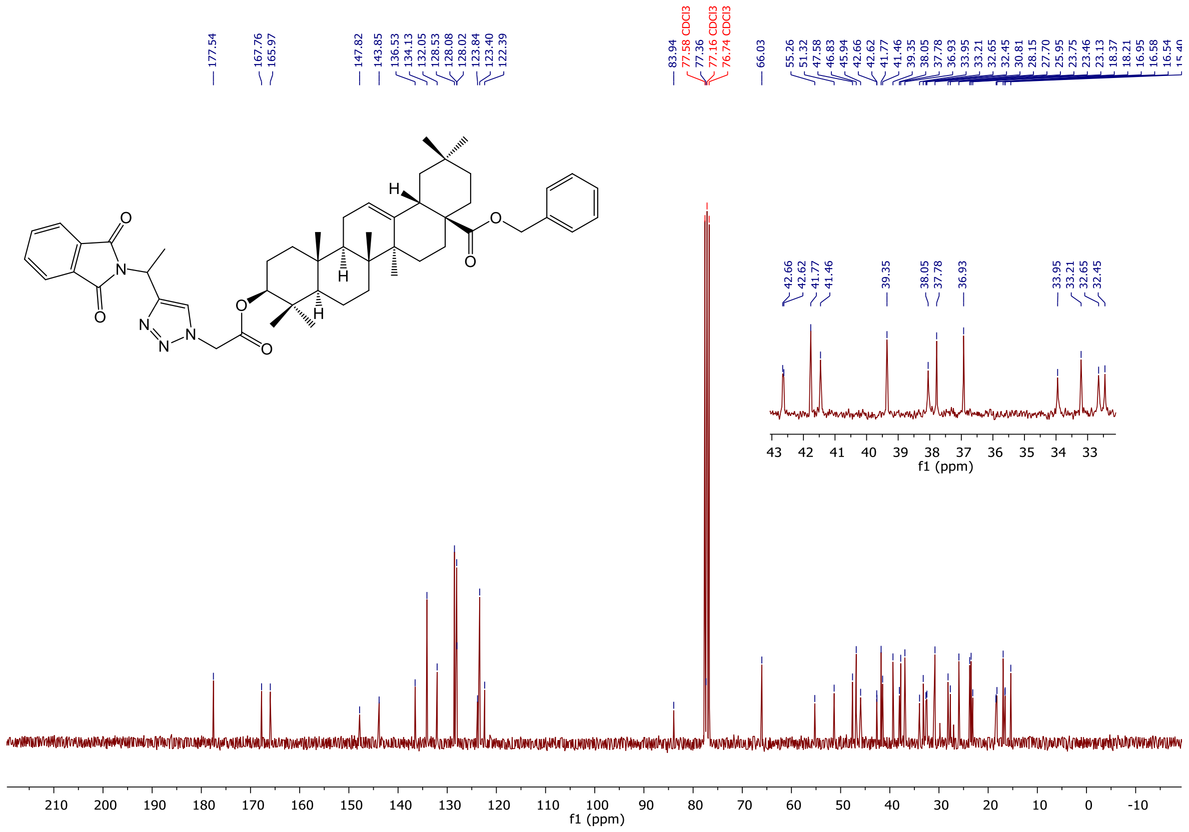

^{13}C NMR spectrum of compound **(18f)**

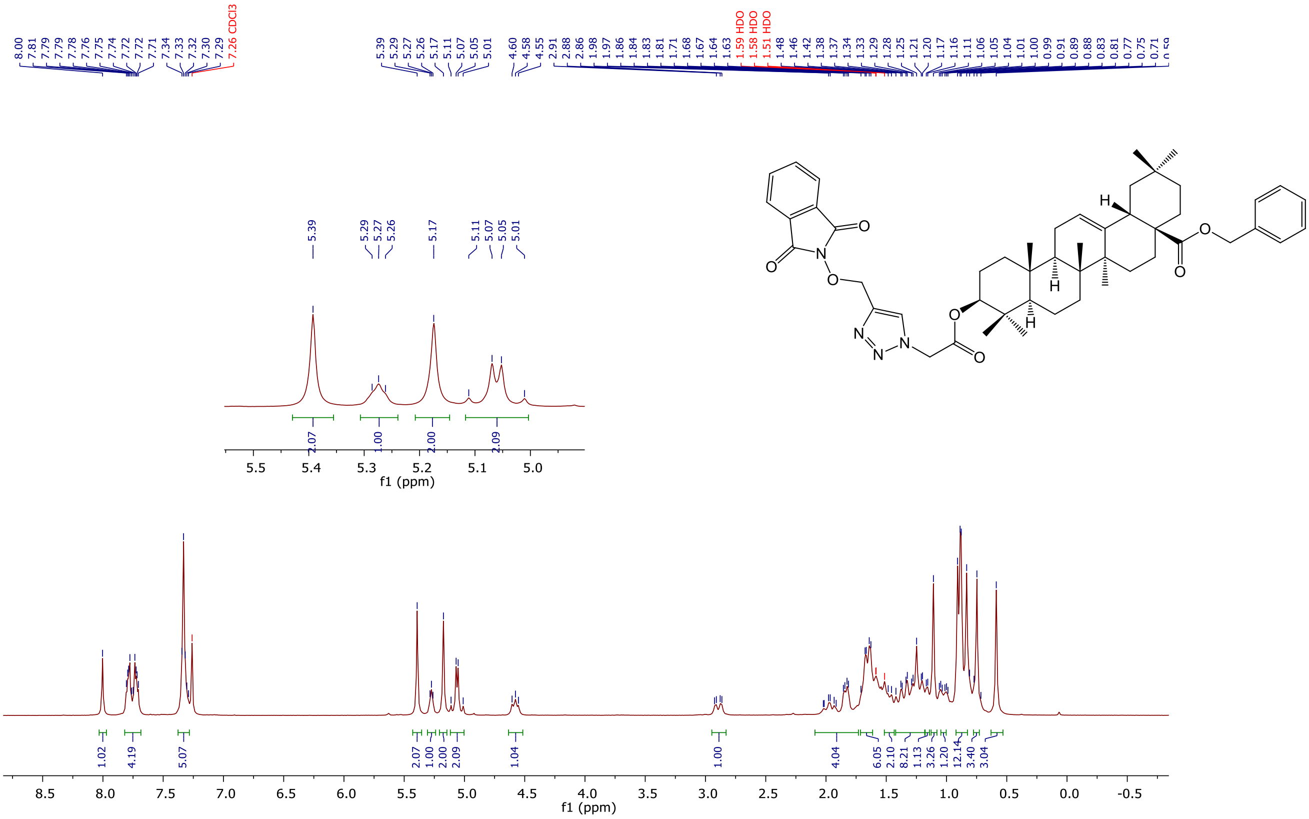

^{1}H NMR spectrum of compound **(18g)**

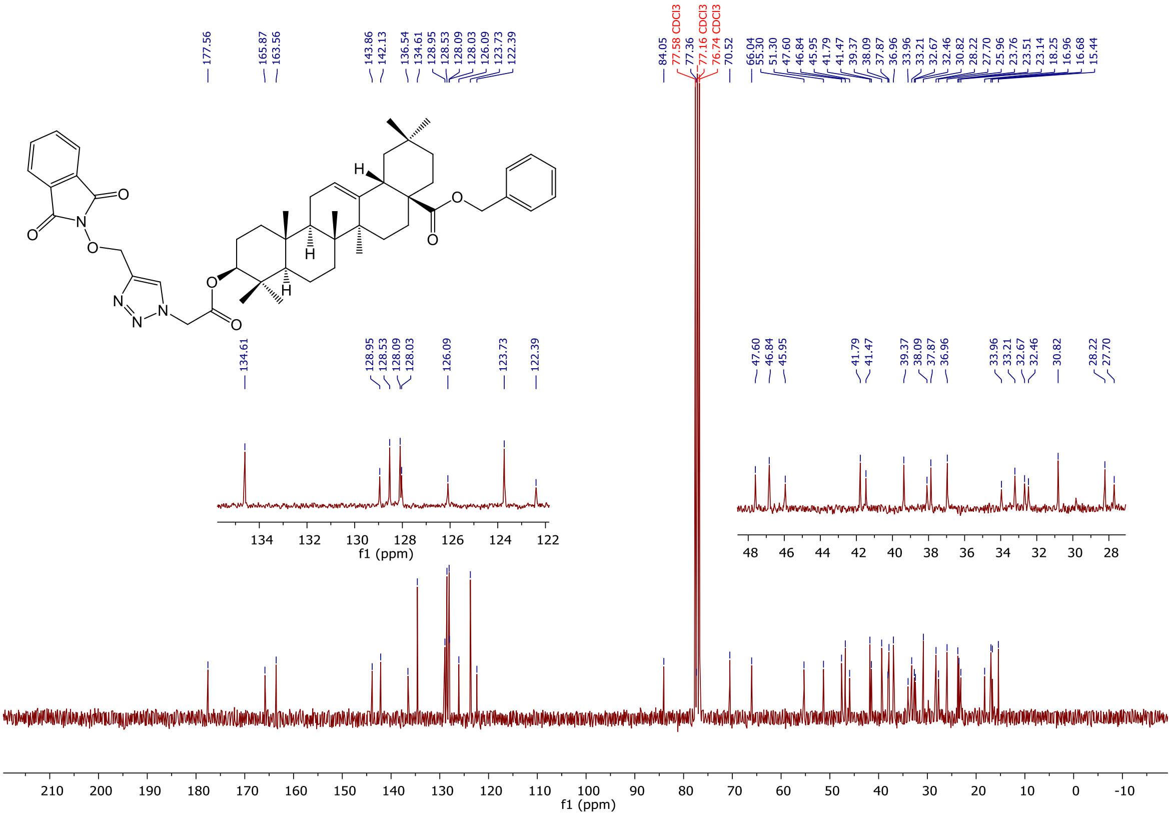

^{13}C NMR spectrum of compound **(18g)**

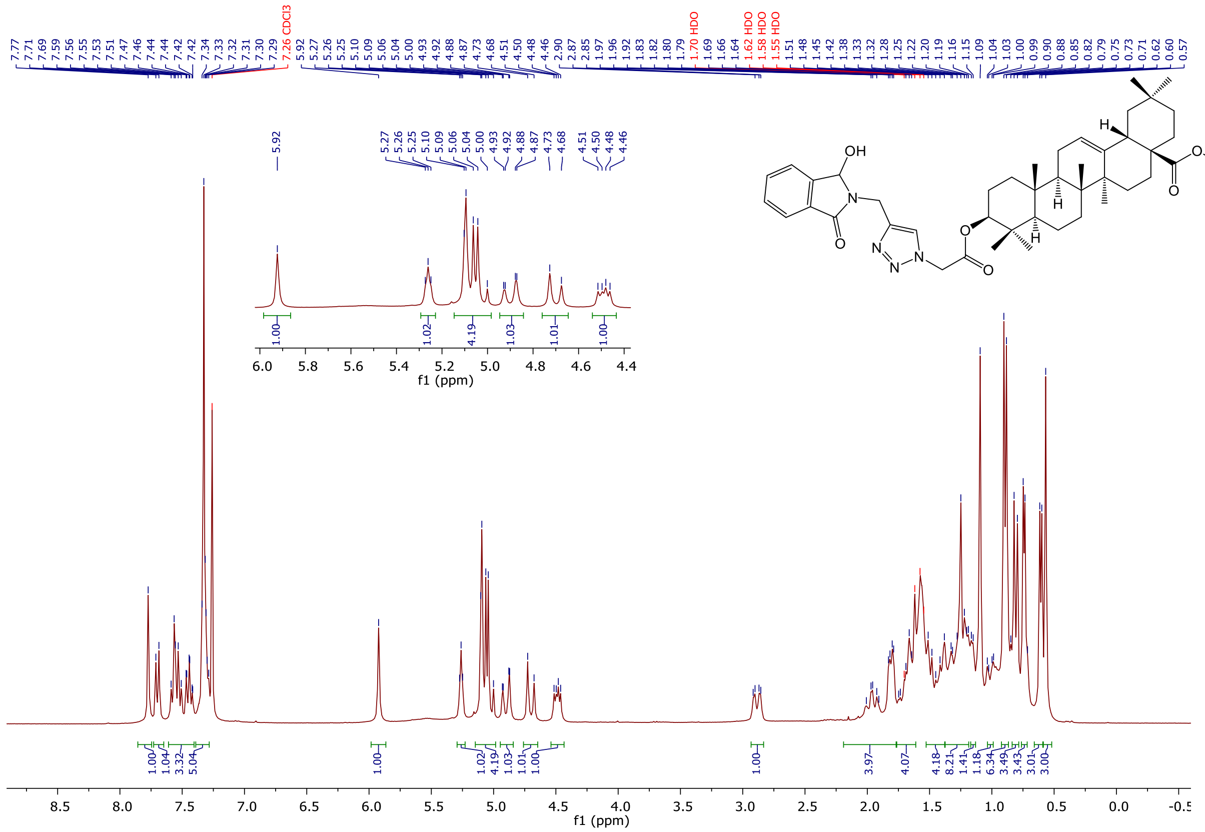

^{1}H NMR spectrum of compound **(18h)**

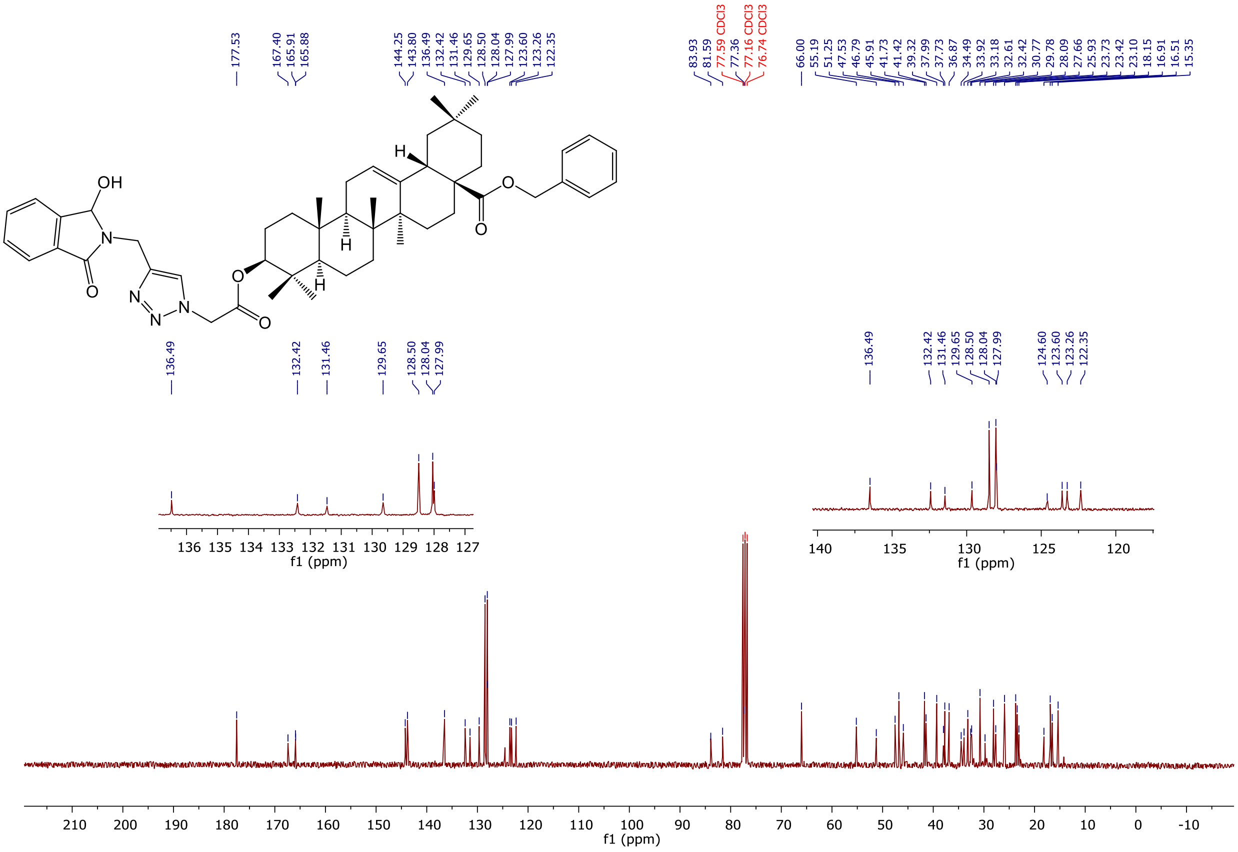

^{13}C NMR spectrum of compound **(18h)**

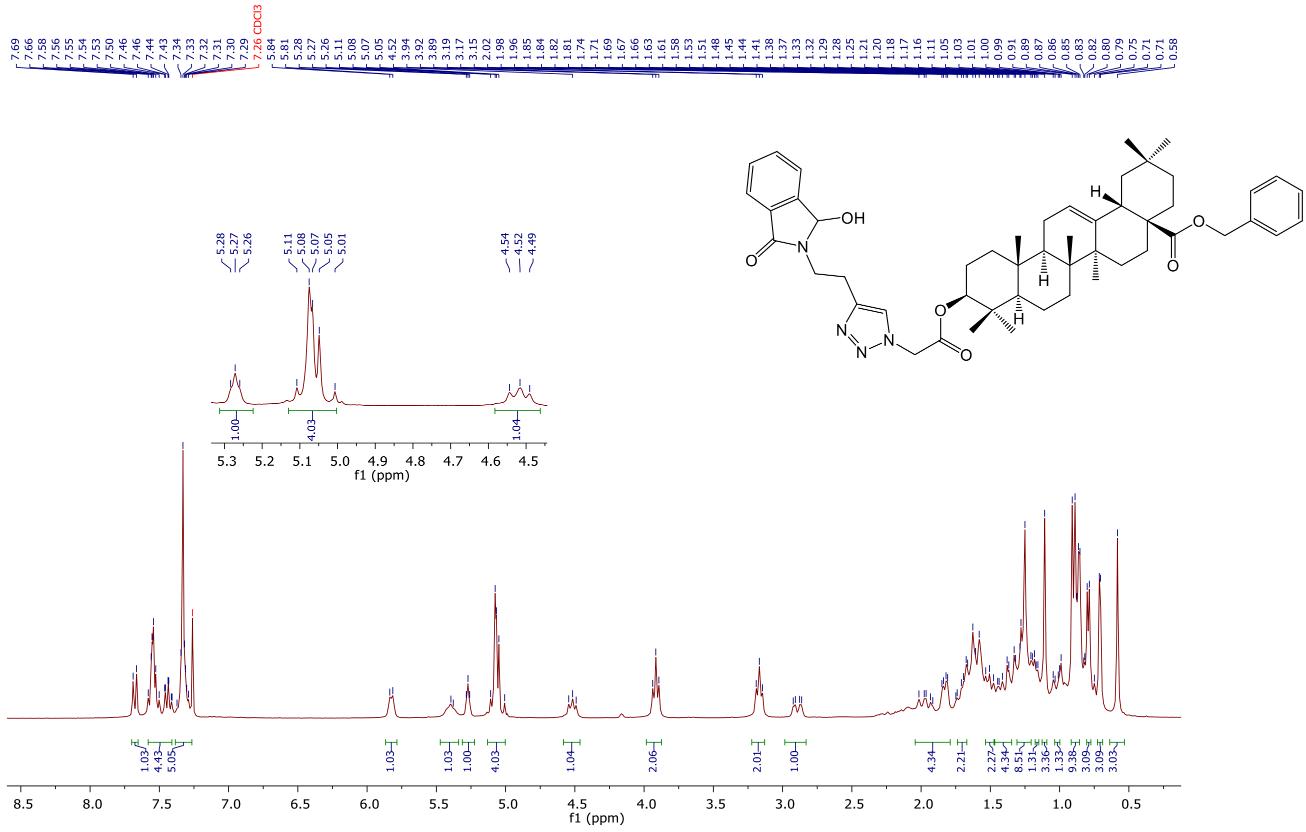

^{1}H NMR spectrum of compound **(18i)**

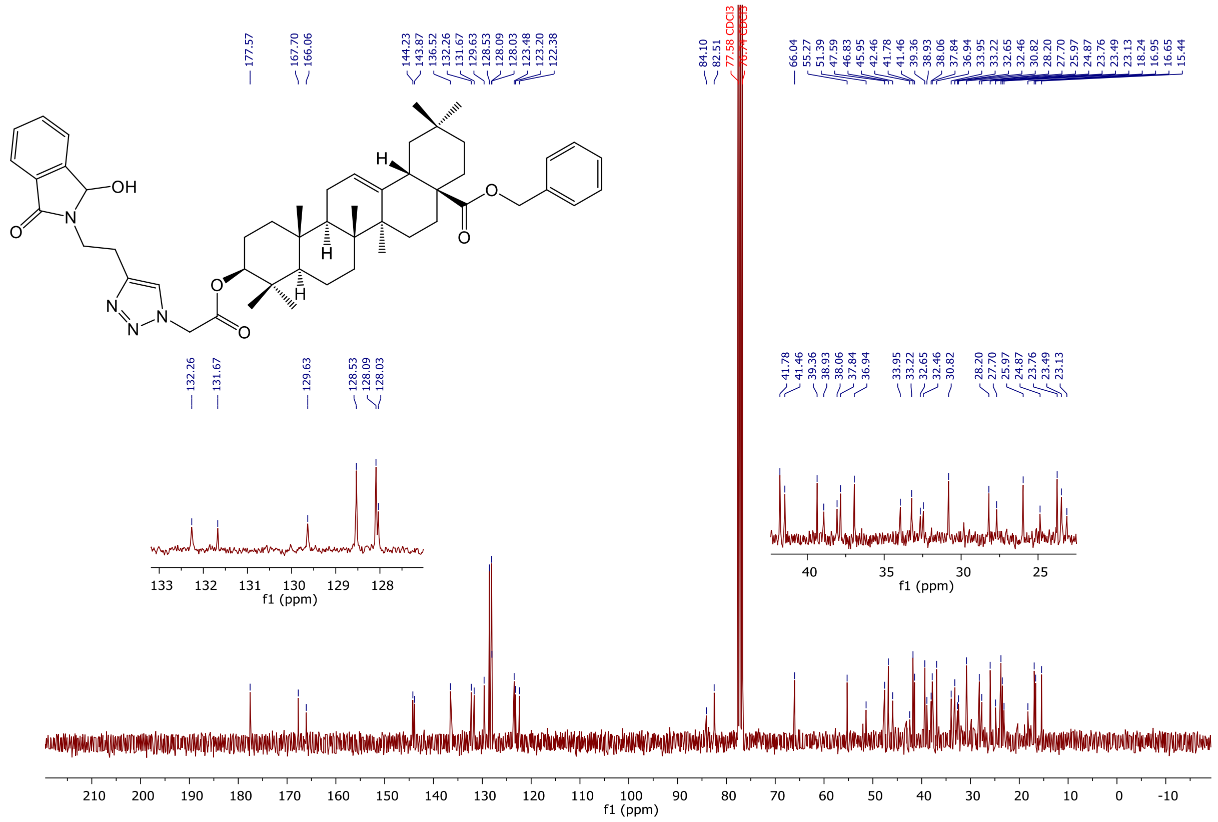

^{13}C NMR spectrum of compound **(18i)**

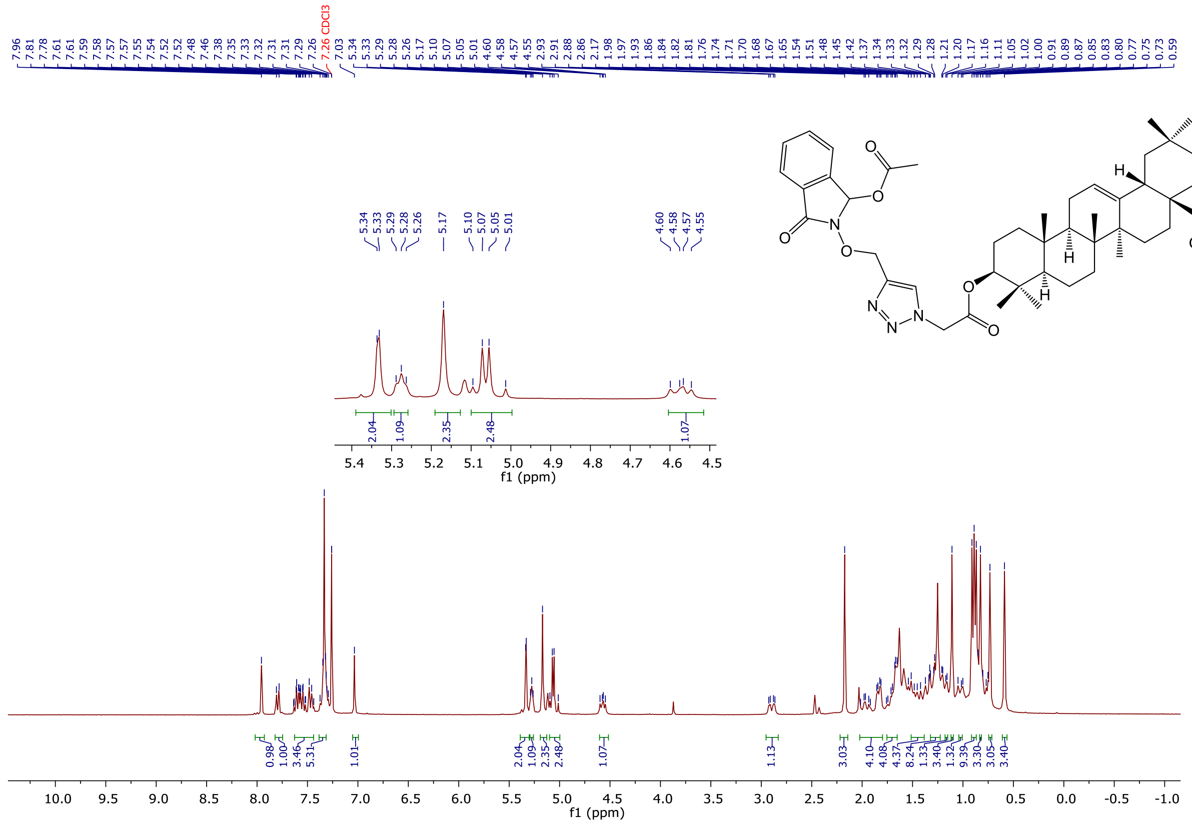

^{1}H NMR spectrum of compound **(18j)**

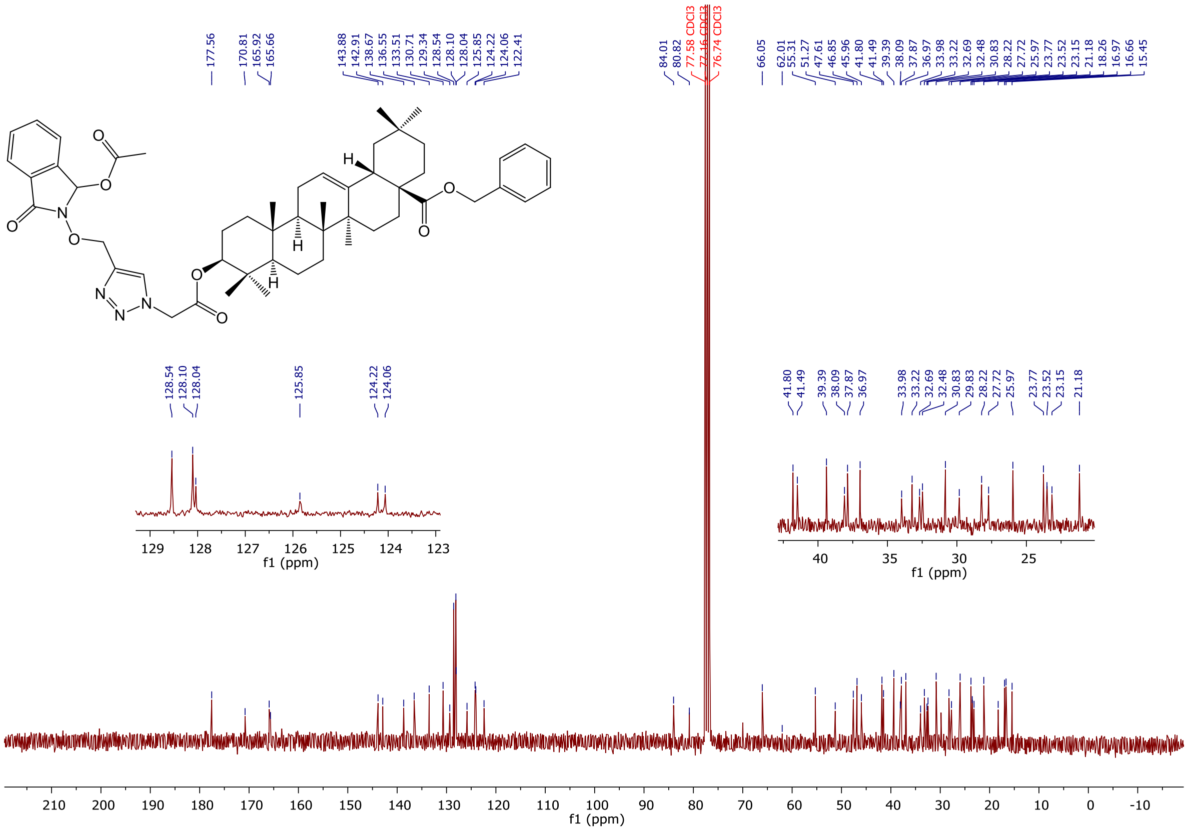

^{13}C NMR spectrum of compound **(18j)**

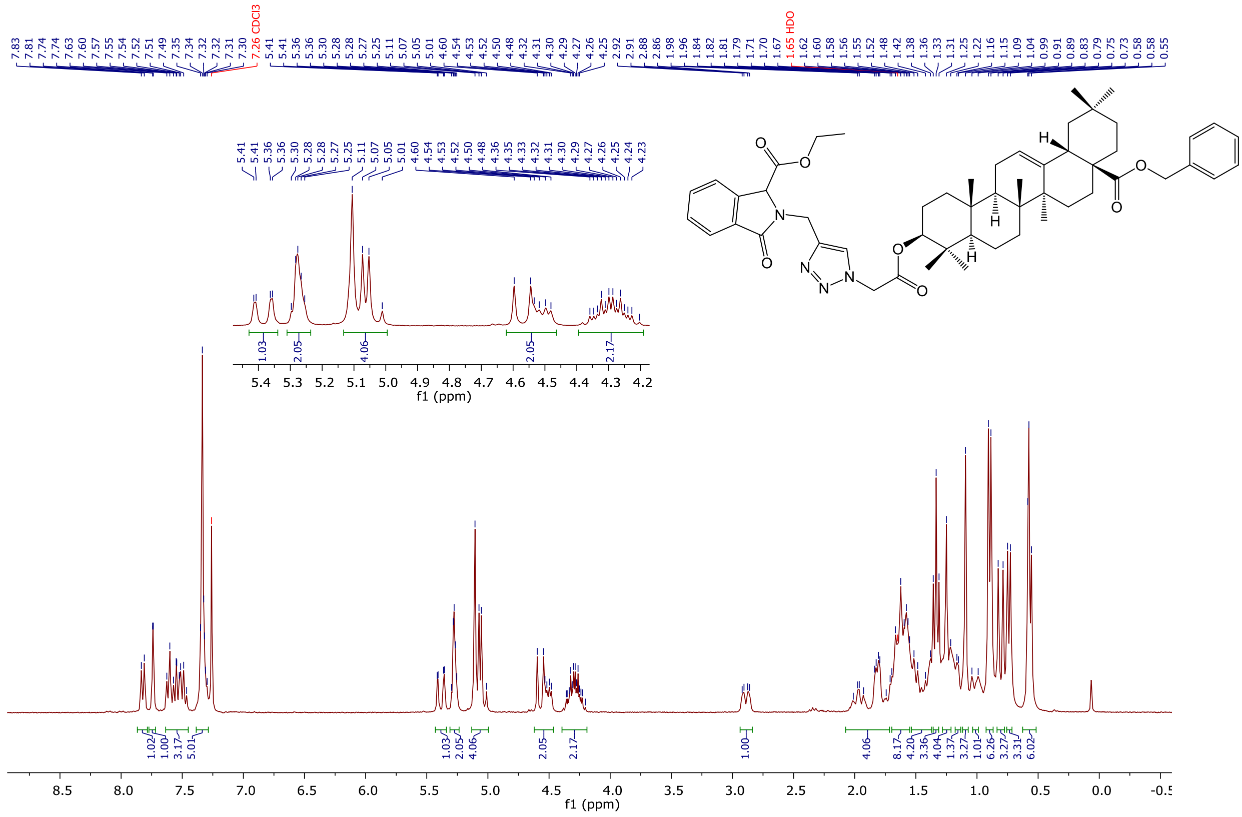

^{1}H NMR spectrum of compound **(18k)**

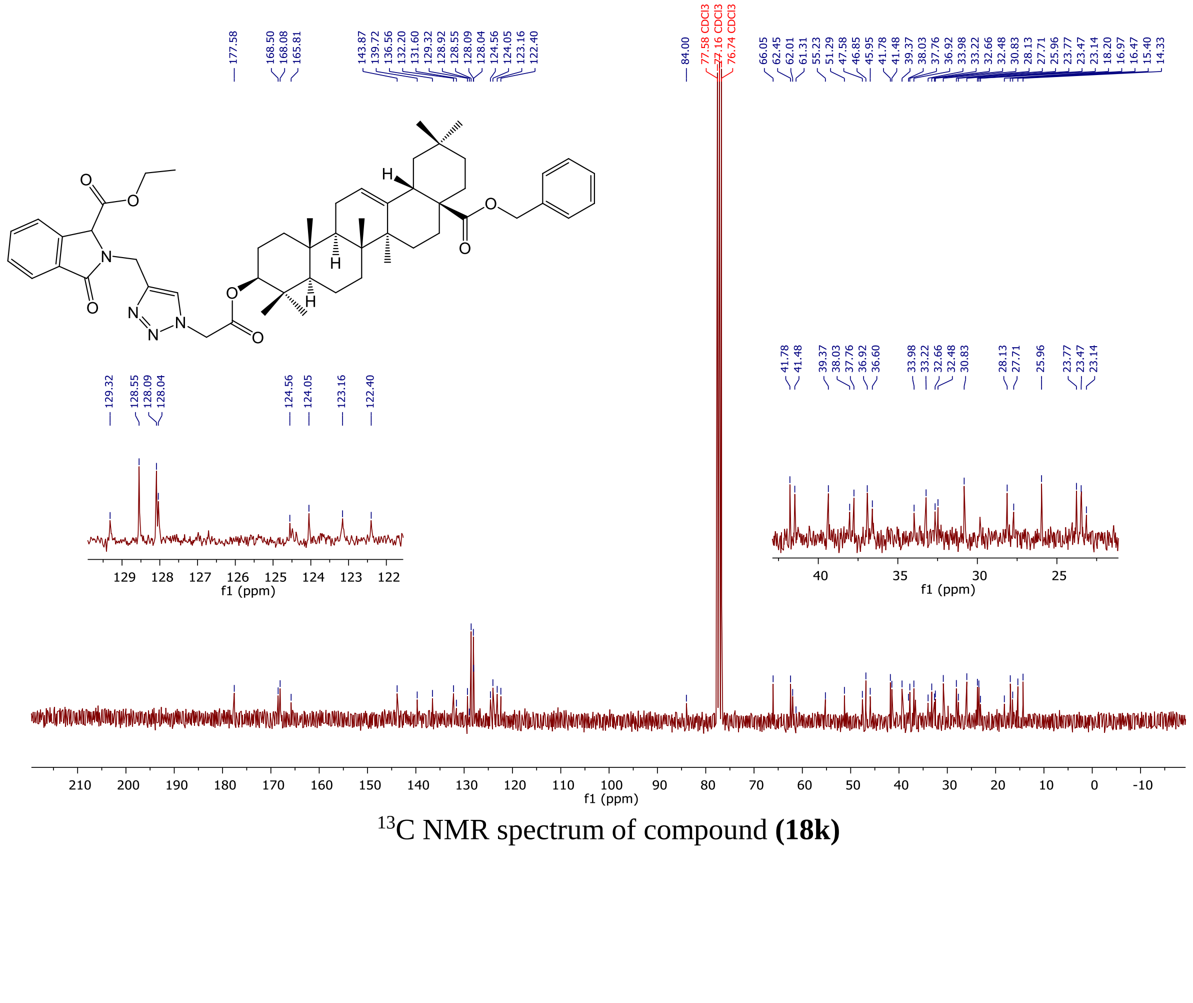

^{13}C NMR spectrum of compound **(18k)**

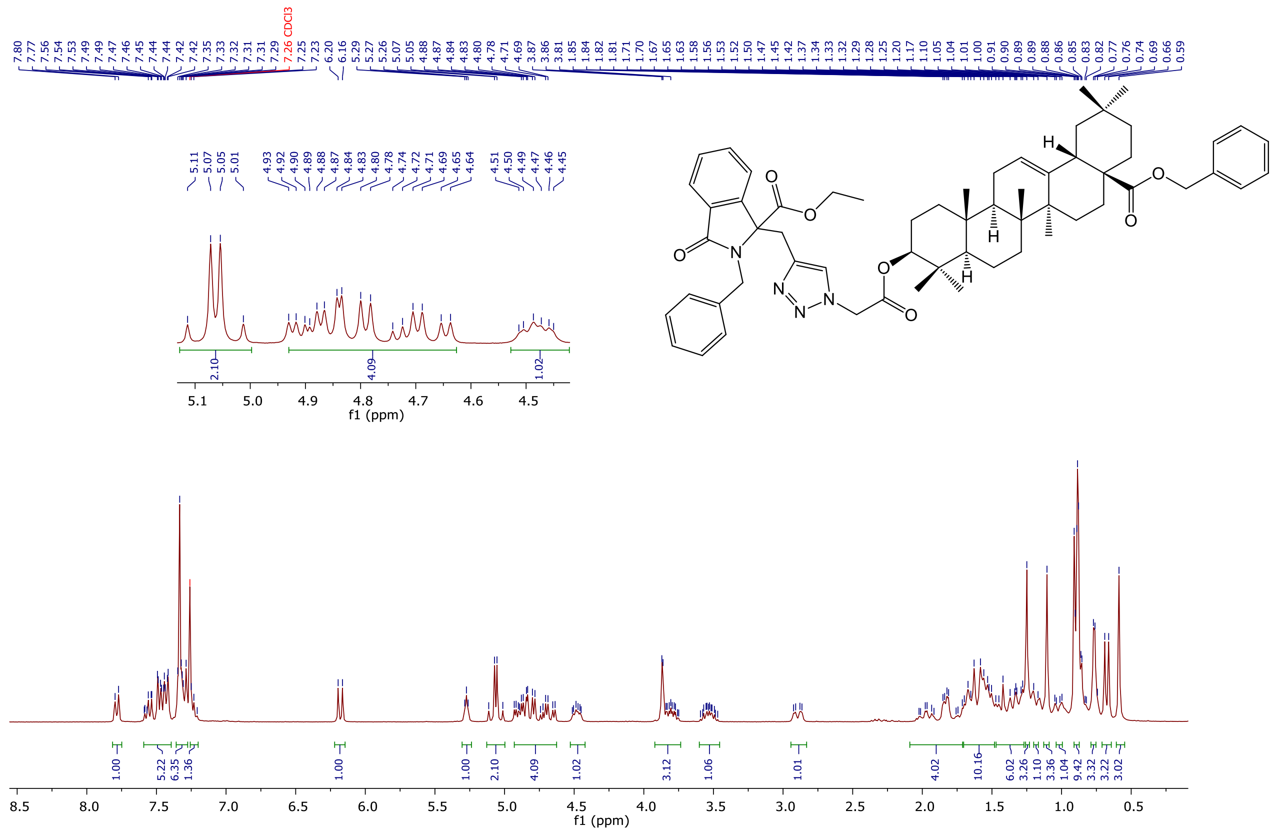

^1H NMR spectrum of compound **(18l)**

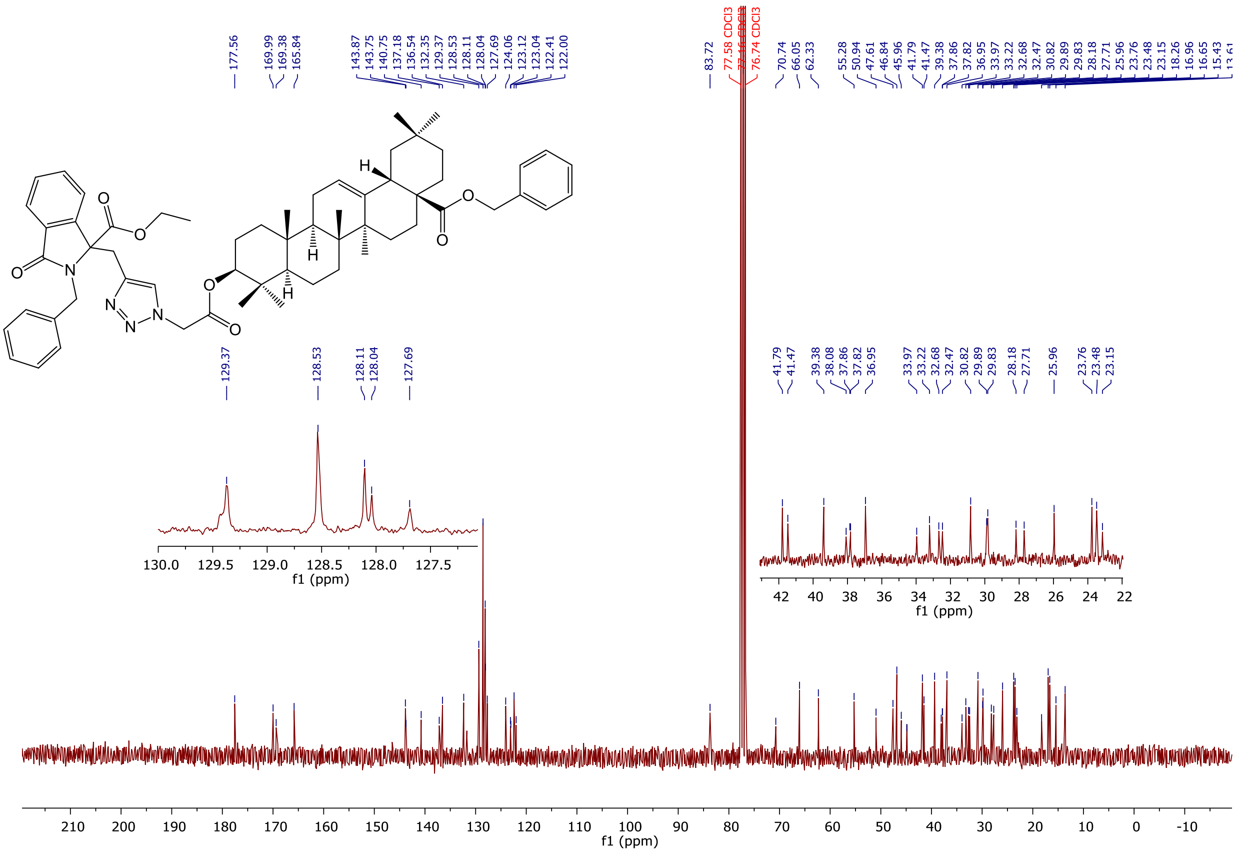

^{13}C NMR spectrum of compound **(18l)**

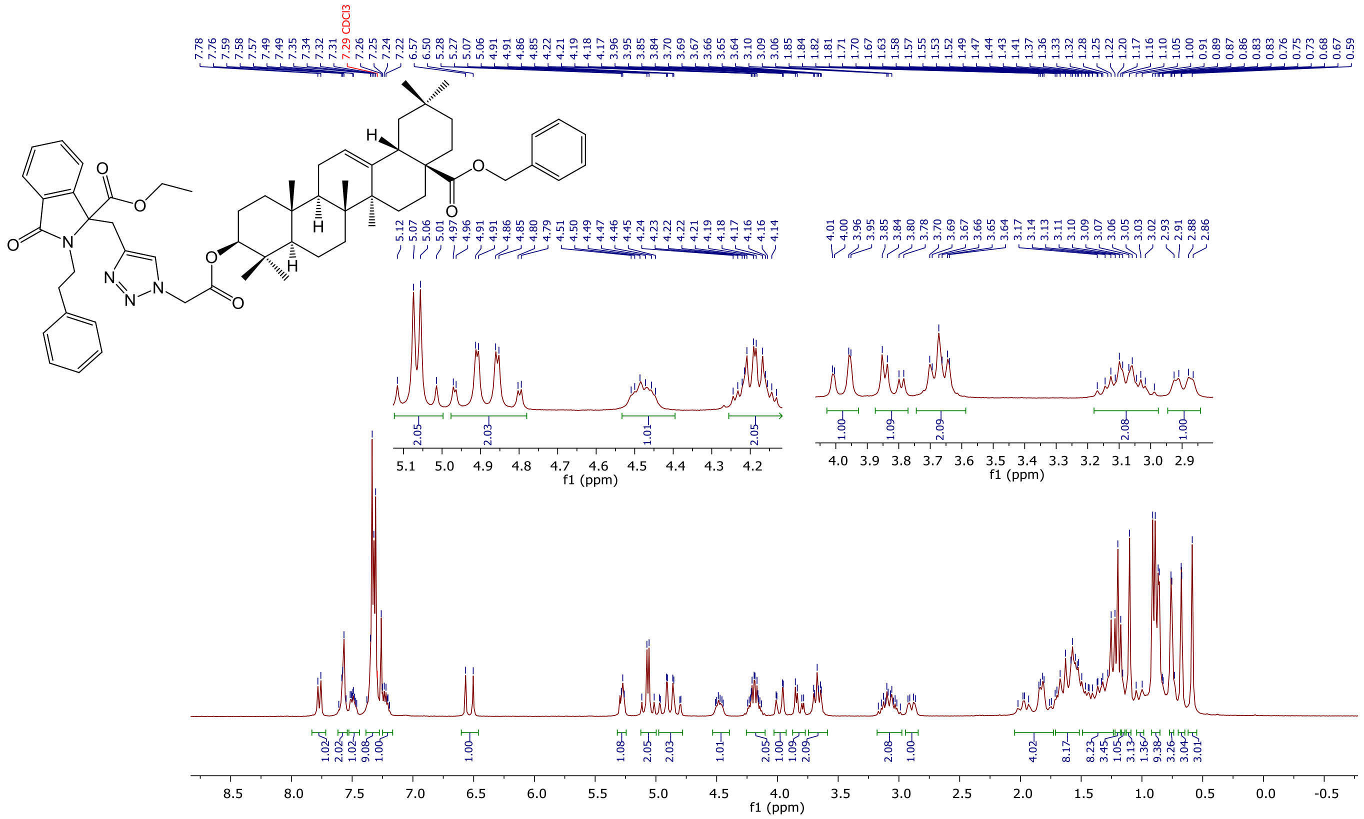

^{1}H NMR spectrum of compound **(18m)**

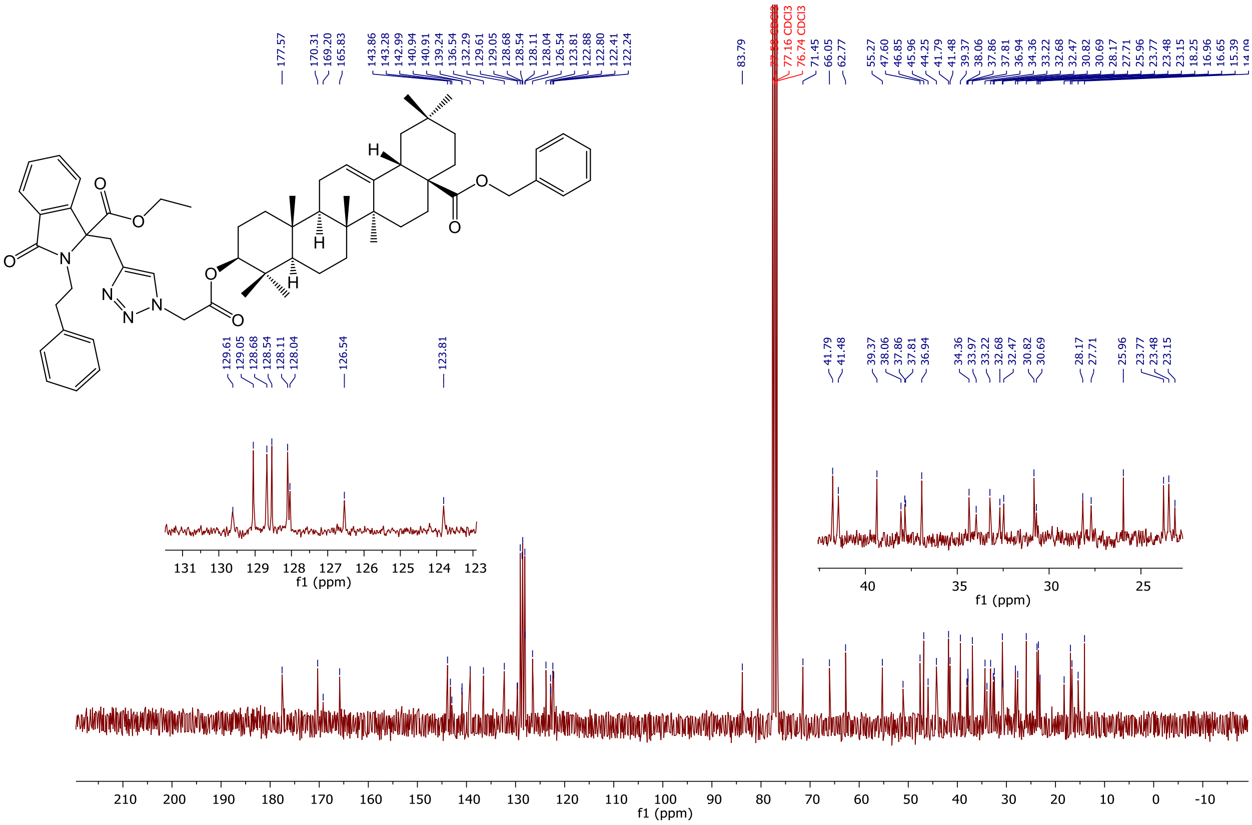

^{13}C NMR spectrum of compound **(18m)**

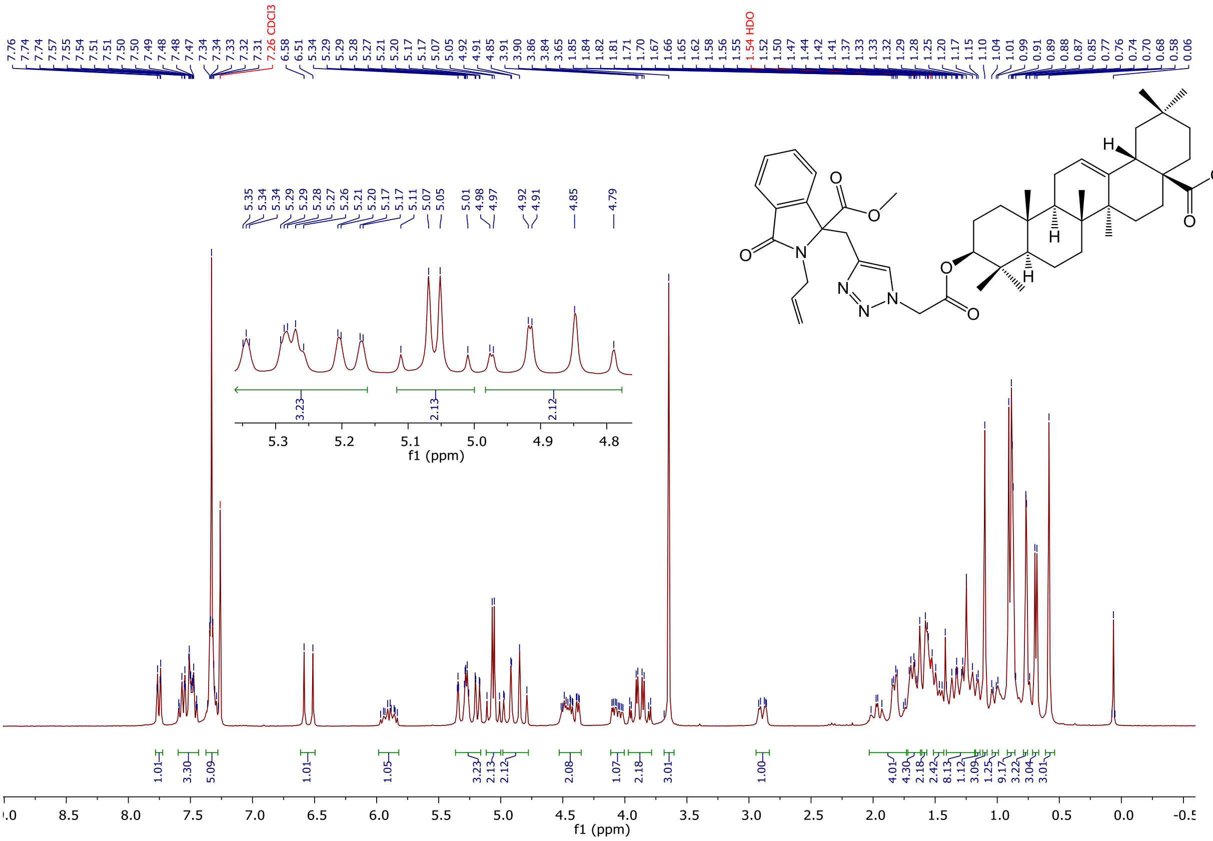

^{1}H NMR spectrum of compound **(18n)**

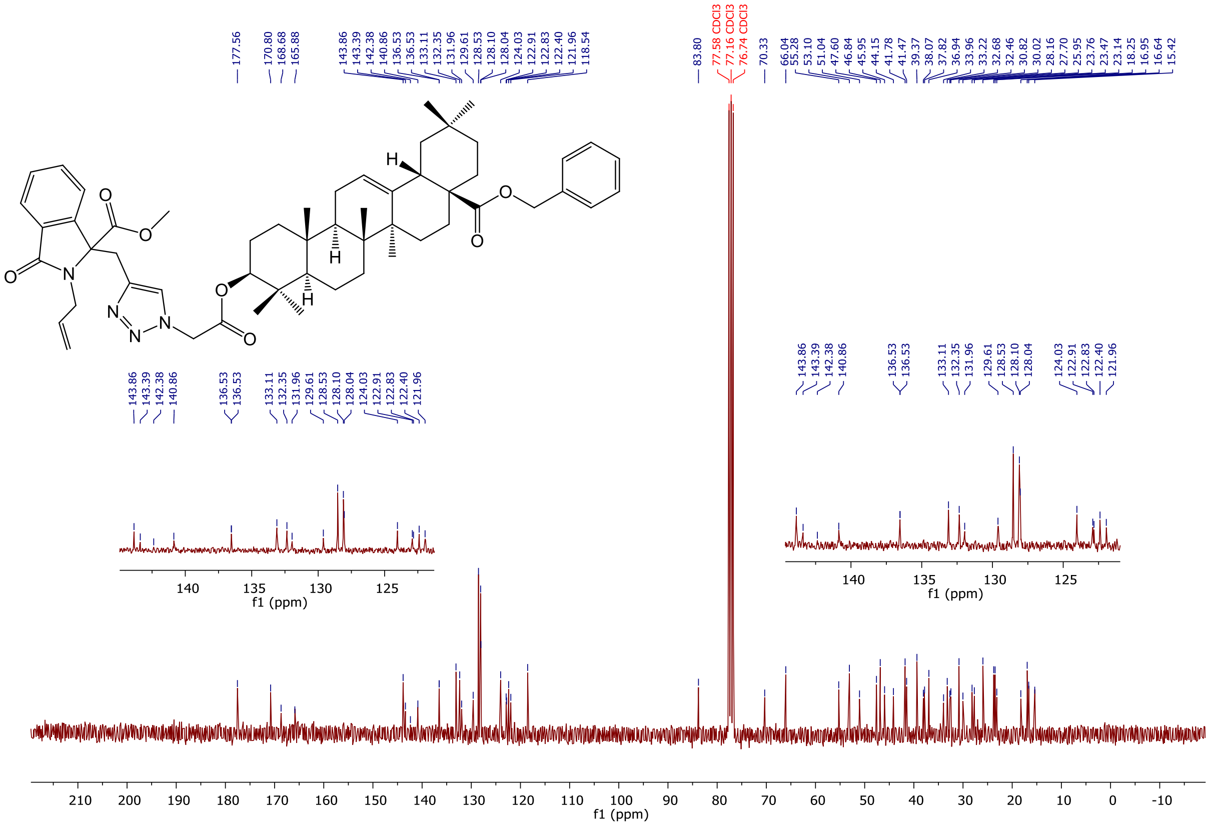

^{13}C NMR spectrum of compound **(18n)**

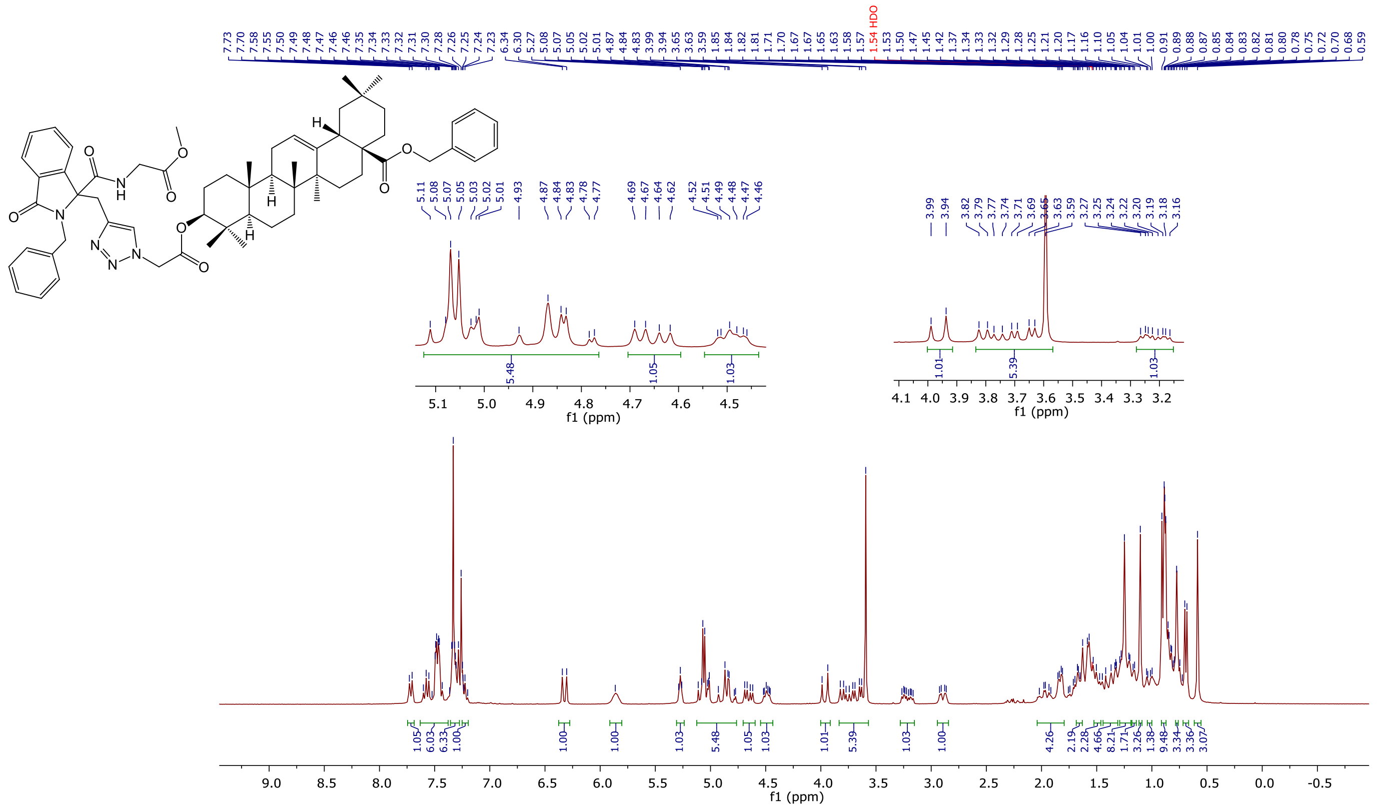

^{1}H NMR spectrum of compound **(18o)**

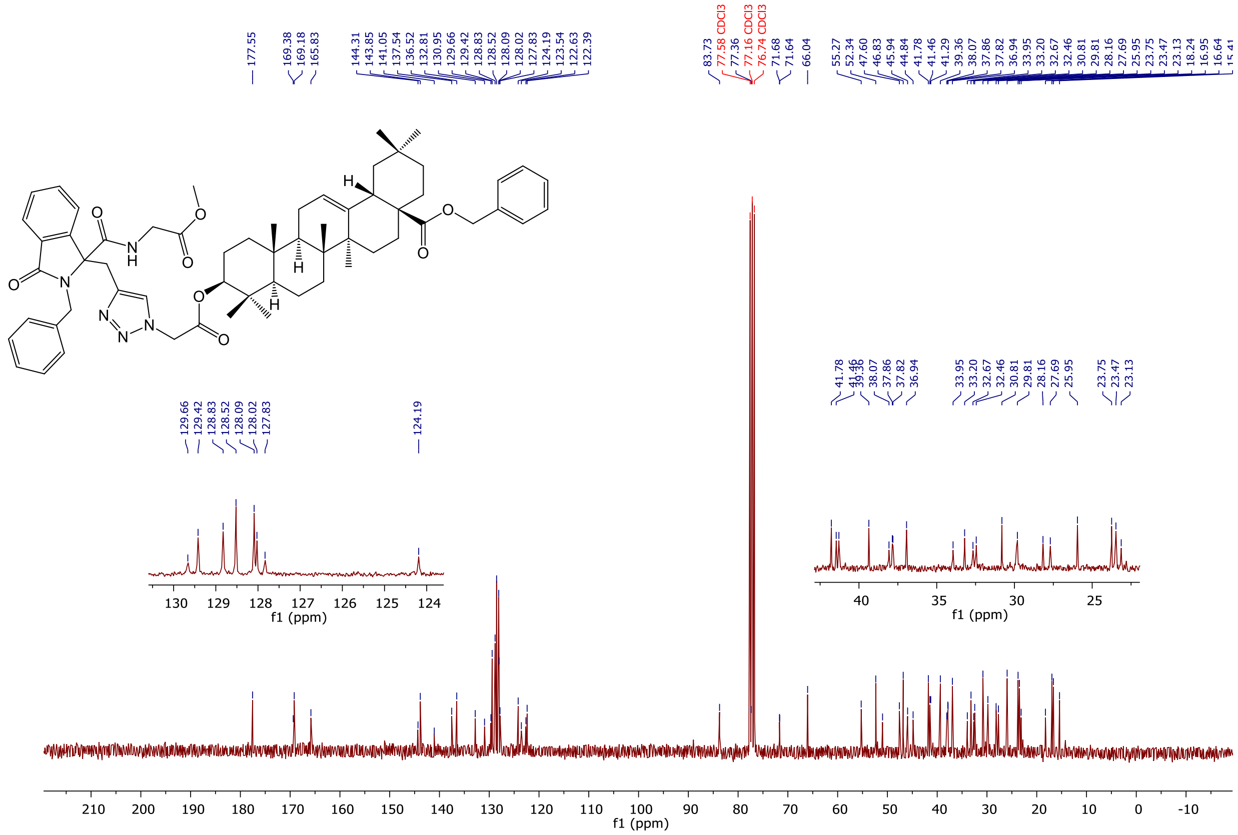

^{13}C NMR spectrum of compound **(18o)**

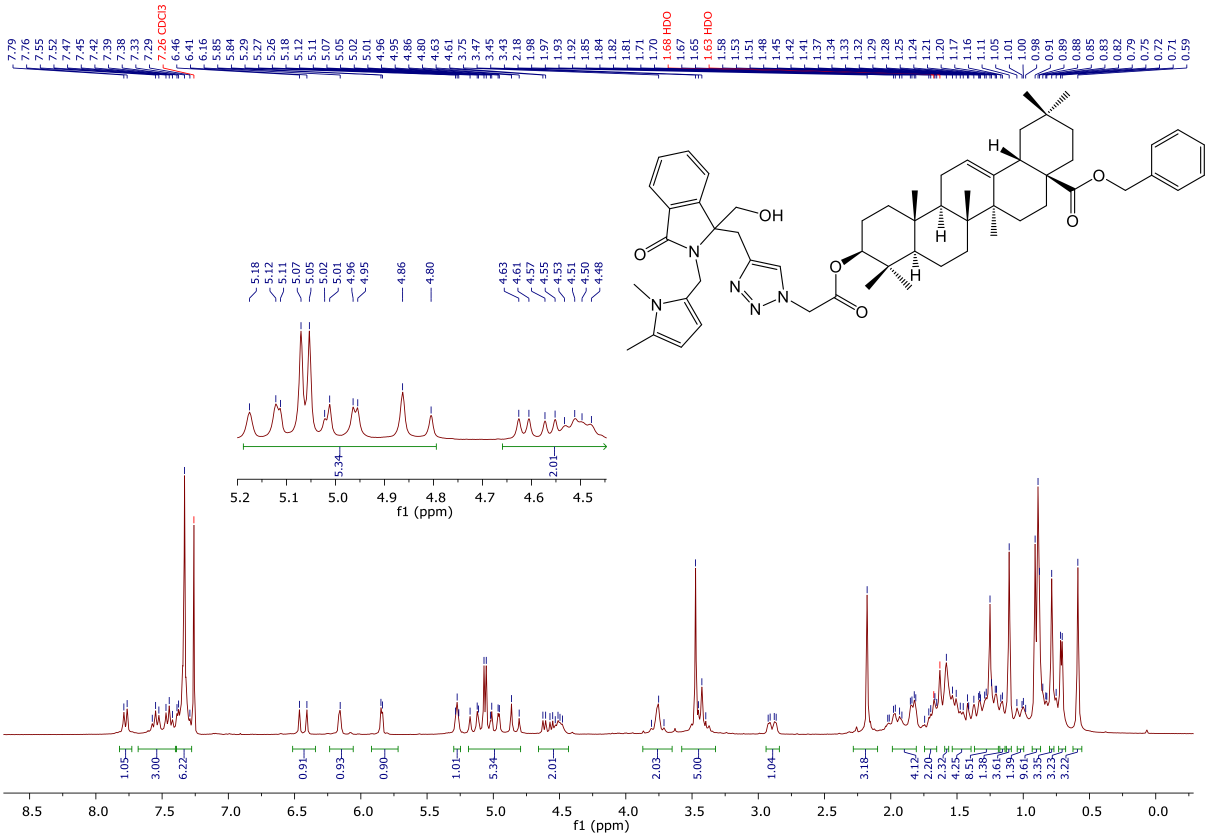

^{1}H NMR spectrum of compound **(18p)**

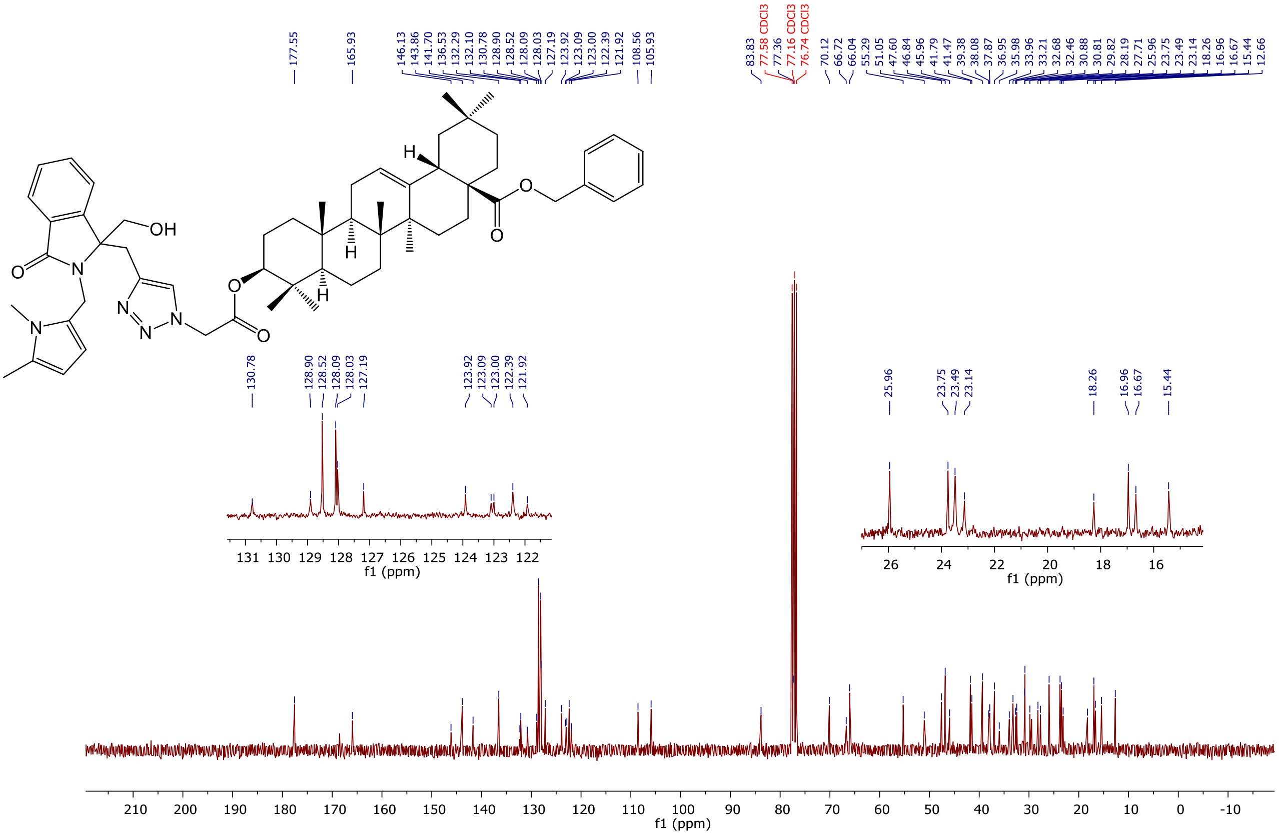

^{13}C NMR spectrum of compound **(18p)**

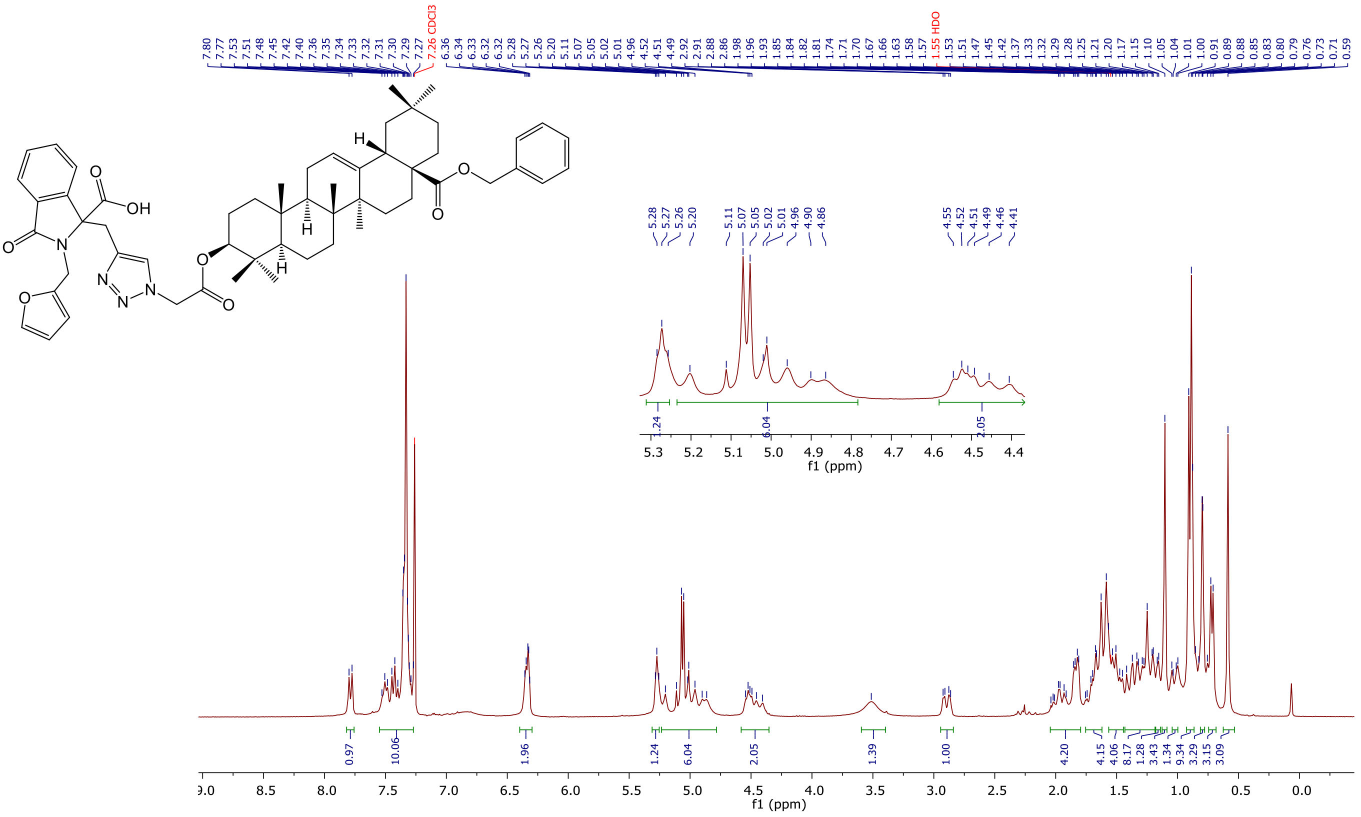

^{1}H NMR spectrum of compound **(18q)**

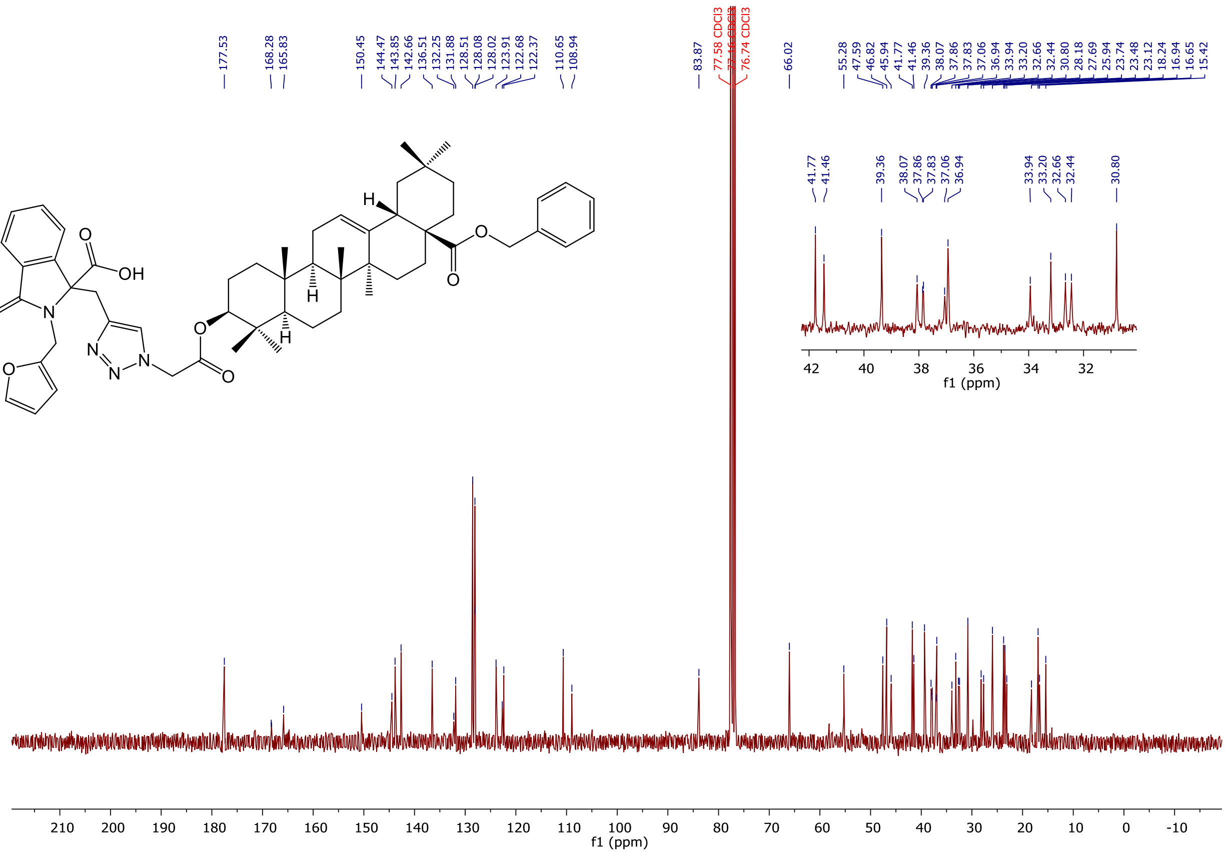

^{13}C NMR spectrum of compound **(18q)**

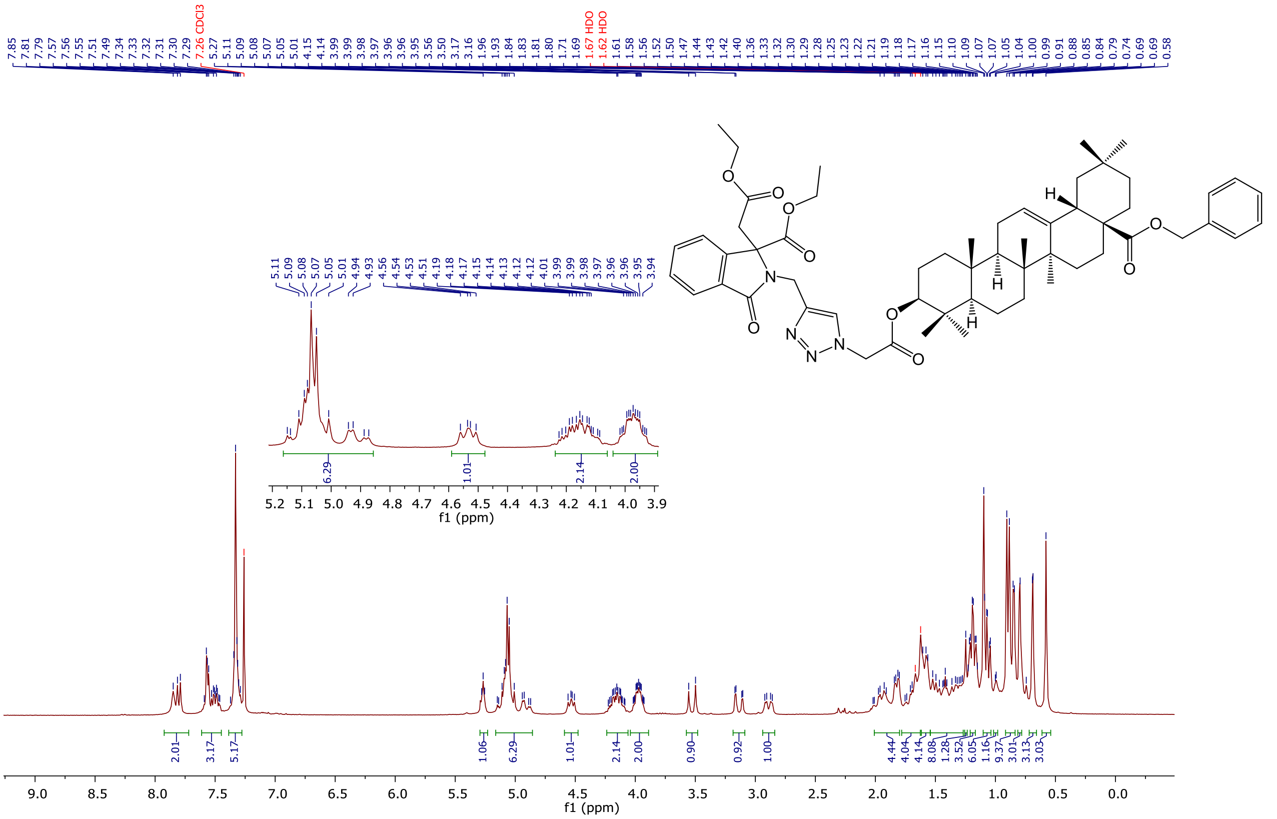

^{1}H NMR spectrum of compound **(18r)**

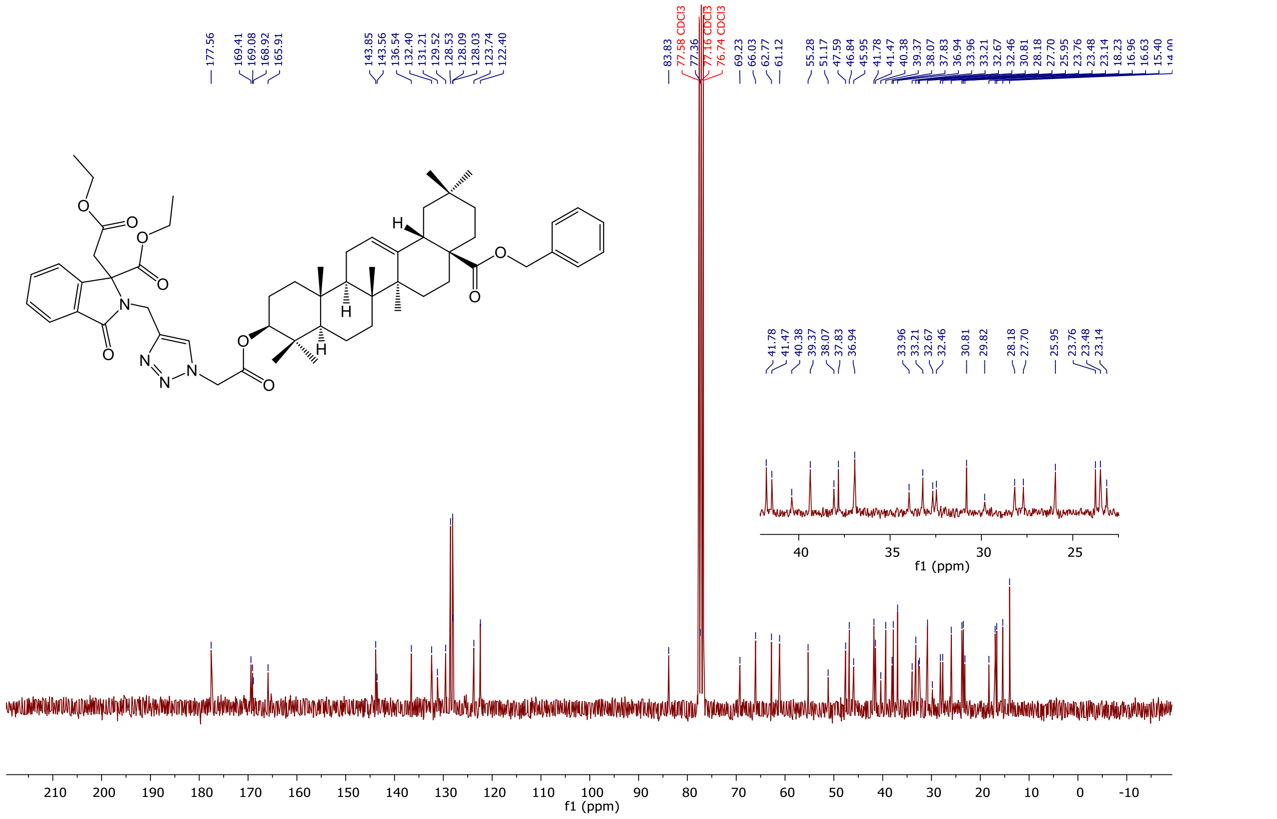

^{13}C NMR spectrum of compound **(18r)**

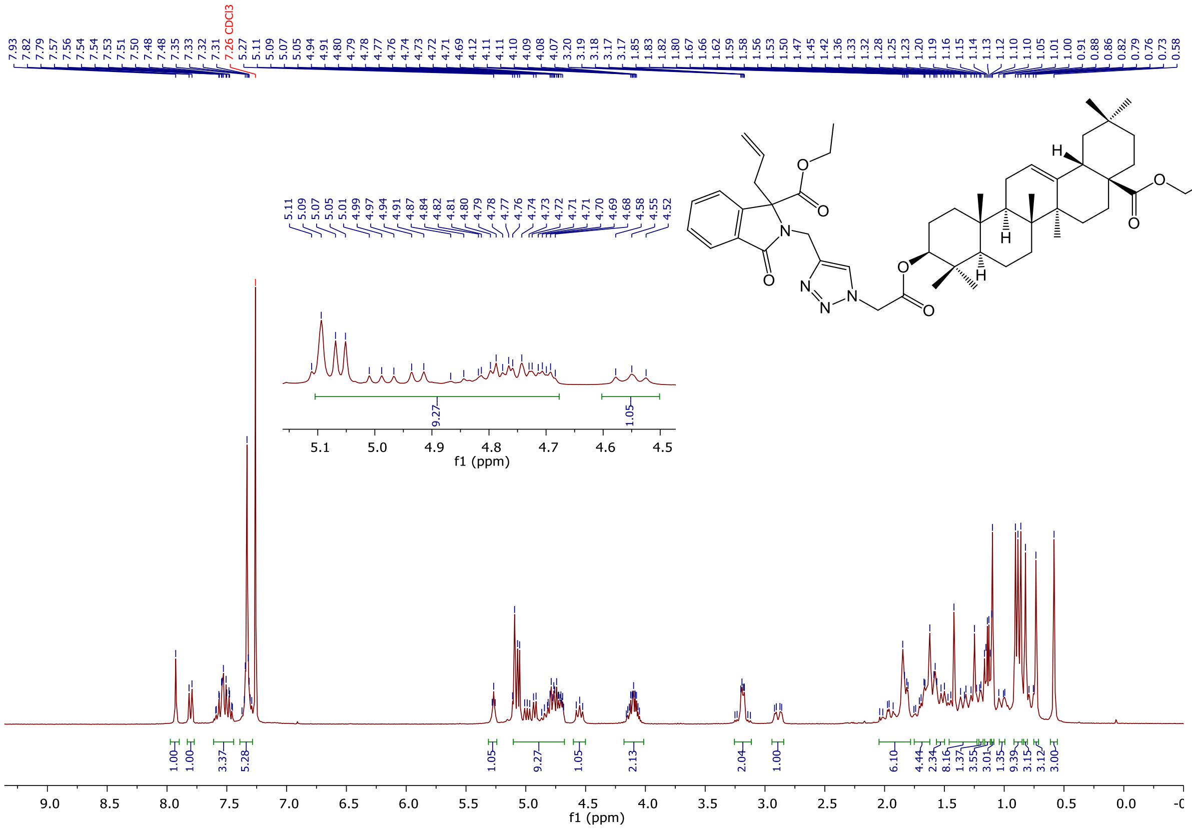

^{1}H NMR spectrum of compound **(18s)**

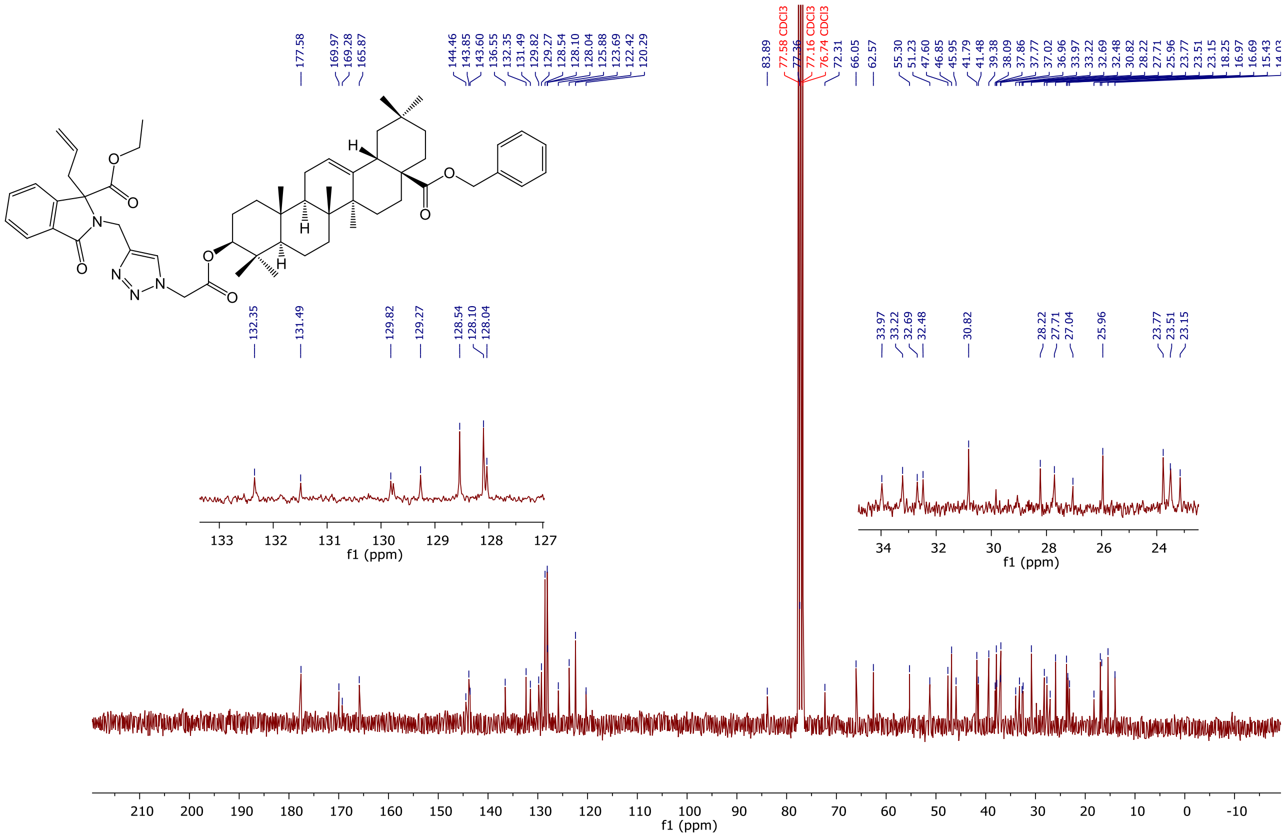

^{13}C NMR spectrum of compound **(18s)**

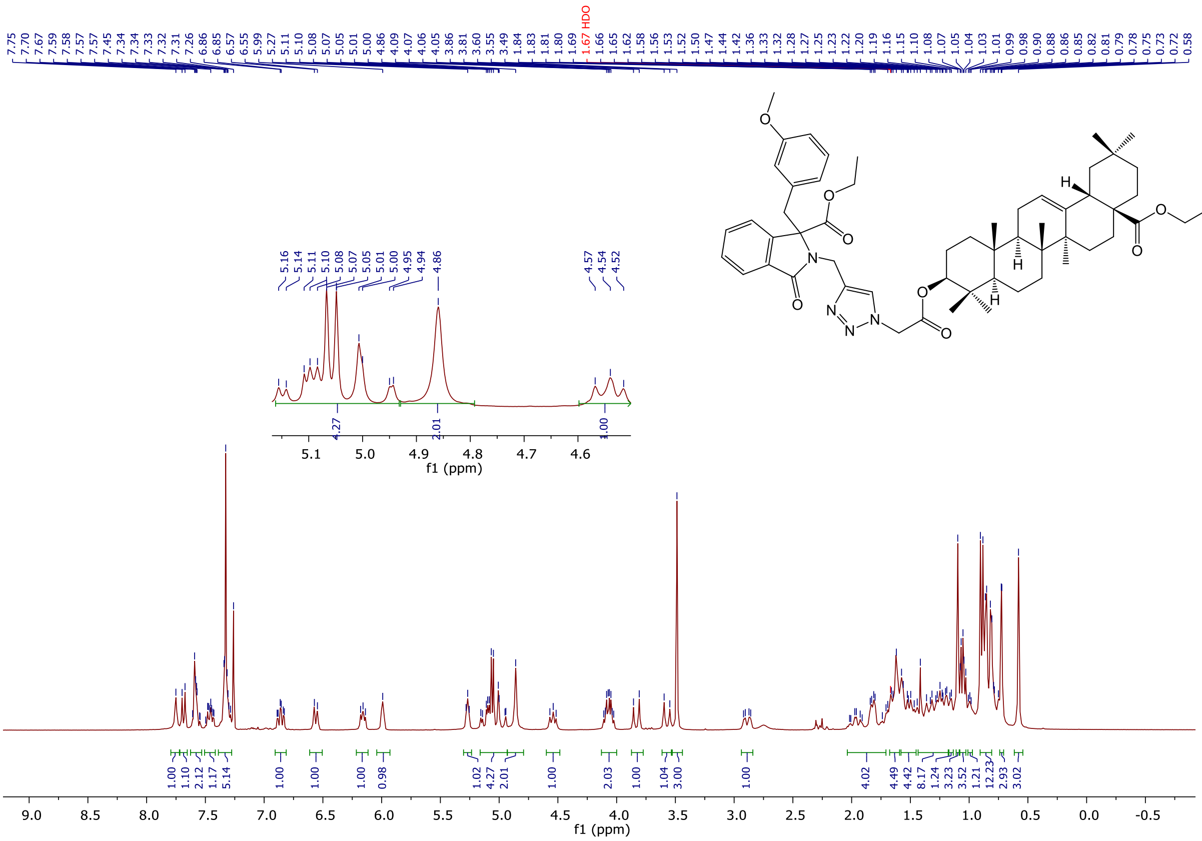

^{1}H NMR spectrum of compound **(18t)**

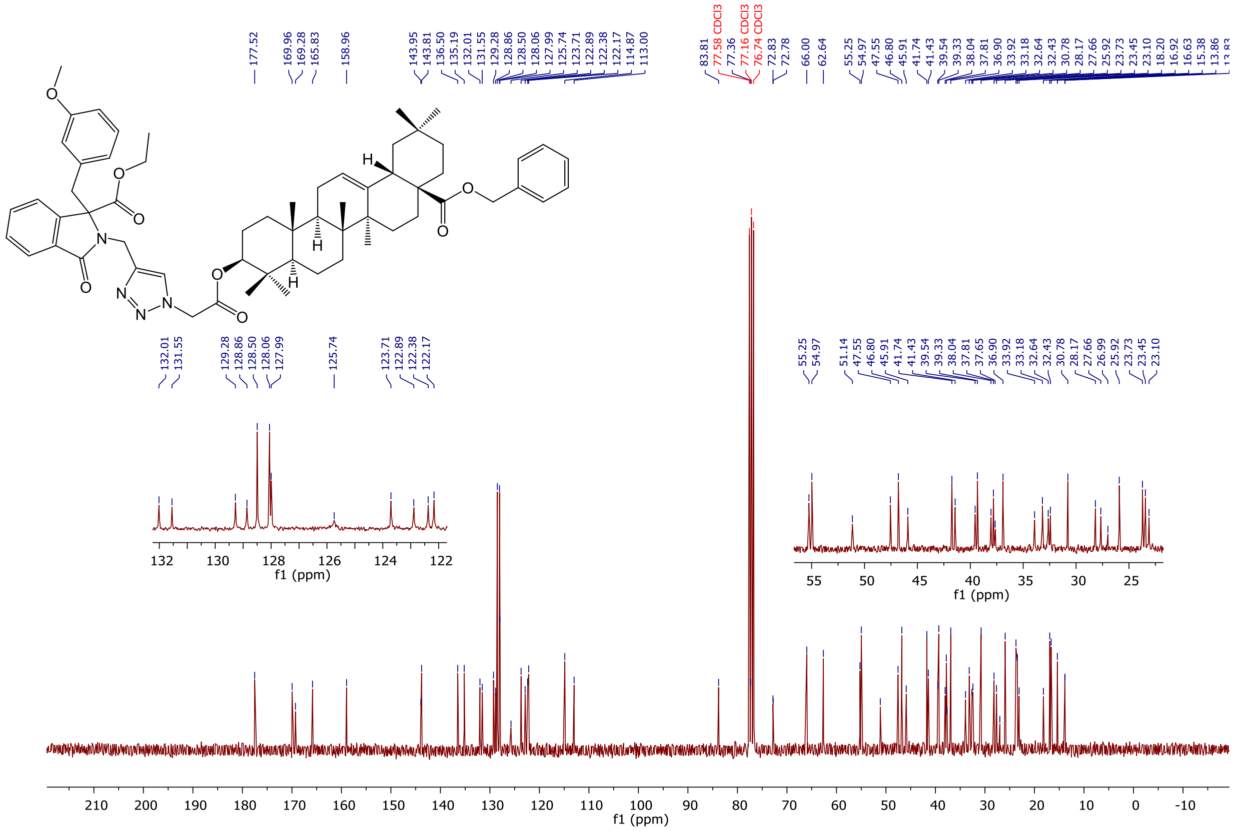

^{13}C NMR spectrum of compound **(18t)**

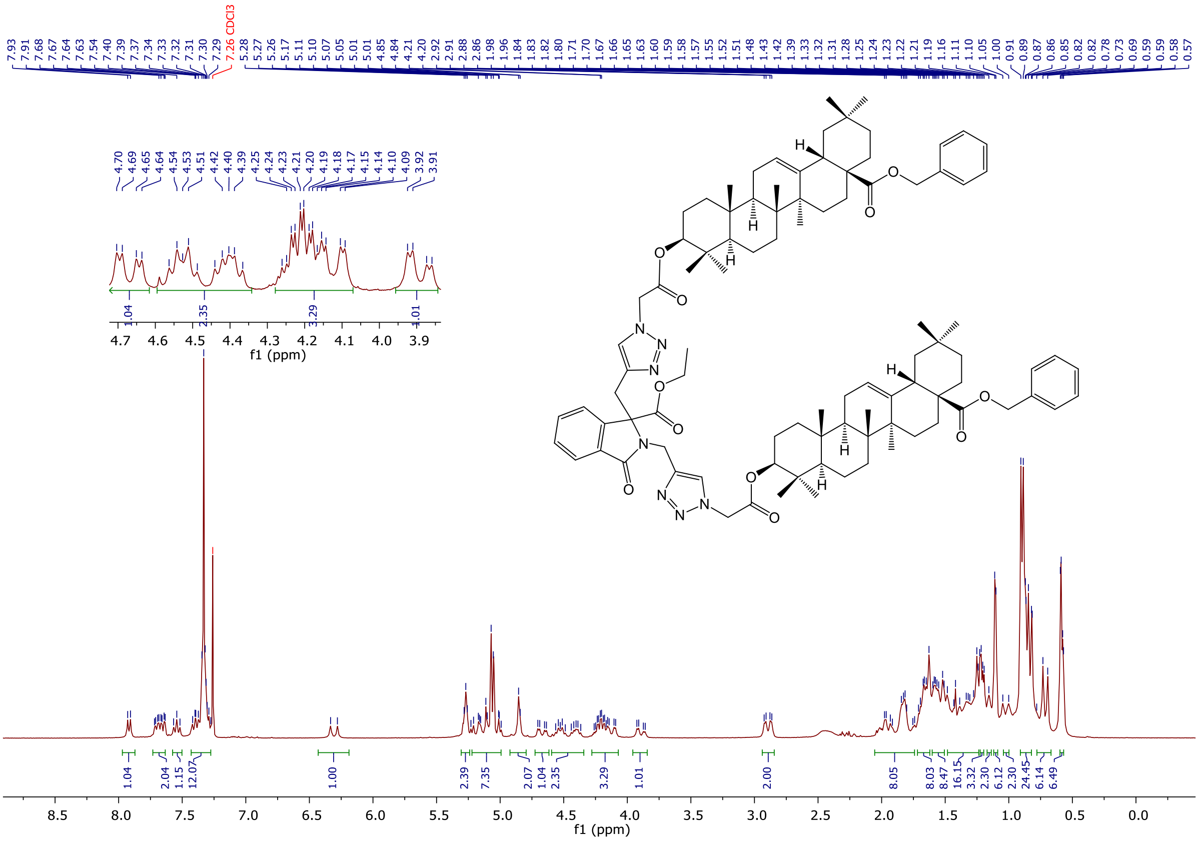

^{1}H NMR spectrum of compound **(18u)**

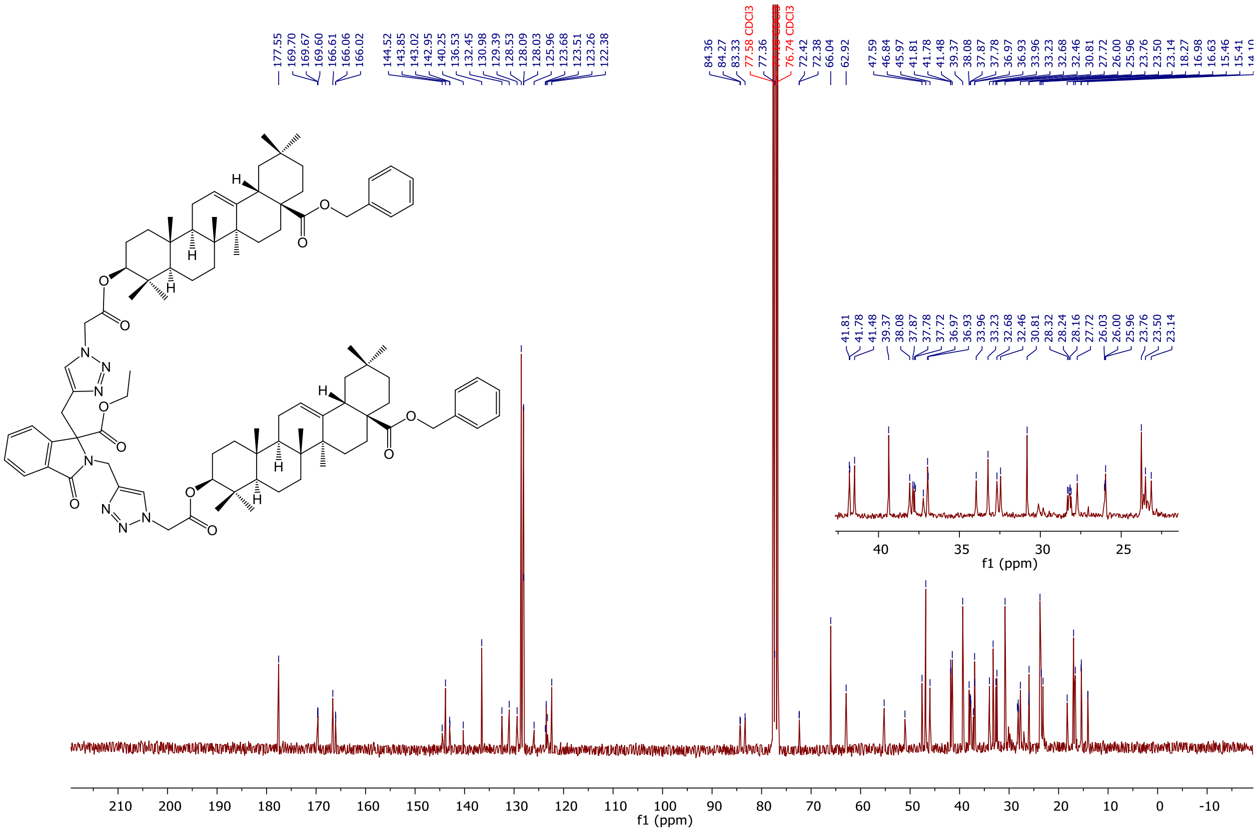

^{13}C NMR spectrum of compound **(18u)**
